# Diverse and tissue-enriched small RNAs in the plant pathogenic fungus, *Magnaporthe oryzae*

**DOI:** 10.1186/1471-2164-12-288

**Published:** 2011-06-02

**Authors:** Cristiano C Nunes, Malali Gowda, Joshua Sailsbery, Minfeng Xue, Feng Chen, Douglas E Brown, YeonYee Oh, Thomas K Mitchell, Ralph A Dean

**Affiliations:** 1 Fungal Genomics Laboratory, Center for Integrated Fungal Research, Department of Plant Pathology, North Carolina State University, Raleigh, NC 27606, USA; 2 Next-Generation Genomics Laboratory, Center for Cellular and Molecular Platform, NCBS-GKVK Campus, Bangalore 560065, India; 3 Department of Plant Pathology, China Agricultural University, Beijing 1000193, China; 4 US DOE Joint Genome Institute, Walnut Creek, CA 94598, USA; 5 Department of Plant Pathology, The Ohio State University, Columbus, OH 43210, USA

## Abstract

**Background:**

Emerging knowledge of the impact of small RNAs as important cellular regulators has prompted an explosion of small transcriptome sequencing projects. Although significant progress has been made towards small RNA discovery and biogenesis in higher eukaryotes and other model organisms, knowledge in simple eukaryotes such as filamentous fungi remains limited.

**Results:**

Here, we used 454 pyrosequencing to present a detailed analysis of the small RNA transcriptome (~ 15 - 40 nucleotides in length) from mycelia and appressoria tissues of the rice blast fungal pathogen, *Magnaporthe oryzae*. Small RNAs mapped to numerous nuclear and mitochondrial genomic features including repetitive elements, tRNA loci, rRNAs, protein coding genes, snRNAs and intergenic regions. For most elements, small RNAs mapped primarily to the sense strand with the exception of repetitive elements to which small RNAs mapped in the sense and antisense orientation in near equal proportions. Inspection of the small RNAs revealed a preference for U and suppression of C at position 1, particularly for antisense mapping small RNAs. In the mycelia library, small RNAs of the size 18 - 23 nt were enriched for intergenic regions and repetitive elements. Small RNAs mapping to LTR retrotransposons were classified as LTR retrotransposon-siRNAs (LTR-siRNAs). Conversely, the appressoria library had a greater proportion of 28 - 35 nt small RNAs mapping to tRNA loci, and were classified as tRNA-derived RNA fragments (tRFs). LTR-siRNAs and tRFs were independently validated by 3' RACE PCR and northern blots, respectively.

**Conclusions:**

Our findings suggest *M. oryzae *small RNAs differentially accumulate in vegetative and specialized-infection tissues and may play an active role in genome integrity and regulating growth and development.

## Background

Over the last decade, several compelling studies have shown that small RNAs are involved in various cellular processes including maintenance of genome integrity, response to DNA damage, biotic stress responses, and regulation of morphological and developmental processes in many organisms [1-5]. Small RNAs are defined as 19 - 40 nucleotides (nt) long molecules derived from double-stranded RNA (dsRNA) or hairpin-structured RNA precursors via activity of proteins such as Dicers, Drosha, and Argonaute (AGO and PIWI) [6,7]. A number of small RNA classes have been described in plants and animals and include small interfering RNAs (siRNAs), microRNAs (miRNAs), and piwi-interacting RNAs (piRNAs) [6,7].

Recent efforts have expanded the eukaryotic small RNA repertoire to include members from the fungal kingdom. In the budding yeasts, *Saccharomyces cerevisiae, S. castellii *and *Candida albicans*, a predominant proportion of the sequenced small RNAs mapped to rRNA loci and transposable elements [8]. In *Aspergillus fumigatus*, a filamentous human pathogenic fungus, analysis of the small transcriptome (15 - 500 nt) revealed that 23% of the sequences corresponded to tRNA or halves of mature tRNA (36 - 39 nt) [9]. Similar tRNA-derived small RNAs have also been reported in human tissues and cell lines (18 - 40 nt) and in *S. cerevisiae *[10-12]. Although the biogenesis of tRNA-derived small RNAs remains to be determined in *A. fumigatus*, Rny1p and Angiogenin have been identified as the ribonucleases responsible for tRNA cleveage in *S. cerevisiae *and in humans, respectively [11,13]. In the filamentous fungus *Neurospora crassa*, recent in depth studies revealed QDE-2-interacting small RNAs (qiRNAs), microRNA-like RNAs (milRNAs) and Dicer-independent siRNAs (disiRNAs) [4,14]. With a length of 20 - 21 nt, qiRNAs are induced by DNA damage, originate from both DNA strands of ribosomal DNA repeat clusters and interact with Argonaute [4]. MilRNAs recruit different components of the RNA silencing protein apparatus, depending on the milRNA precursor locus, to generate distinct small RNAs varying from 19 and 25 nt. DisiRNAs have a peak distribution length of around 21/22 nt and are not dependent on Dicer proteins for biogenesis [4,14]. In the zygomycete *Mucor circinelloides*, a novel class of small RNAs, named exonic-siRNAs (ex-siRNAs), was recently reported. These RNA-dependent RNA Polymerase 1 and Dicer-like 2 derived molecules target and likely regulate mRNAs of genes from which they are produced [15]. Although knowledge of the small transcriptome in fungi is emerging, in depth studies examining differential accumulation, particularly in plant pathogenic fungi such as the rice blast fungus, *Magnaporthe oryzae*, have not been reported.

*M. oryzae *is the primary plant pathogenic threat to rice production around the world as well as to other economically important cereal crops including wheat and barley [16-18]. Infection is initiated by attachment of the conidium to the leaf surface. Following germination, the germ tube tip develops into a specialized structure, called the appressorium, from which emerges a penetration peg that directly penetrates into plant tissues [19]. The fungus is highly tractable in the laboratory and appressorium formation can be induced by various chemical treatments or by a hydrophobic surface [20-22]. Because of the considerable risk to food security and experimental malleability, this plant pathogenic fungus has been the focus of intensive investigation, including comprehensive genome studies [23]. Several transcription data sets have been obtained including ESTs, SAGE, and MPSS that provide support for many predicted coding genes as well as confirm the presence of non-coding transcripts [21,24]. Recently, we identified a new class of methylguanosine-capped and polyadenylated small RNAs (< 200 nt) (CPA-sRNAs) from mycelia tissue in *M. oryzae *[25].

The genome sequence combined with expression data has greatly enabled characterization of many protein-coding genes [26,27] as well as the molecular machinery required for RNA silencing [28,29]. However, detailed knowledge of the small RNA (15 - 40 nt) repertoire remains to be elucidated. This information represents an important foundation to further understand small RNA biogenesis, their roles in *M. oryzae *growth, development, and pathogenesis and possibly open new avenues for future disease control. Here, we applied 454 sequencing technology to elucidate and characterize the small RNA transcriptome (15 - 40 nt) of mycelia and appressoria of *M. oryzae*.

## Results

### Small RNAs characterization

To examine the small RNA catalog in the plant pathogenic fungus *M. oryzae*, we isolated RNA ranging from ~ 15 - 40 nt and constructed small RNA libraries from mycelia and appressoria tissues, corresponding to vegetative and specialized-infection stages, respectively (see Material and Methods section). After 454 pyrosequencing, a total of 318, 454 and 343, 303 raw sequences were obtained from mycelia and appressoria, respectively (Table 1). Following linker removal, sequences ≥ 16 nt long were mapped to *M. oryzae *genome version 6. Recent literature on RNA sequencing highlights several posttranscriptional modifications and RNA editing in many organisms, including *M. oryzae*, resulting in imperfect alignment of RNAs to the reference genome [25,30]. Therefore, in addition to small RNAs (100% coverage; 100% identity) that mapped perfectly to the genome, we analyzed an imperfectly matching data set (80% coverage; 80% identity) in order to further investigate these modifications. A total of 77, 880 and 39, 268 sequences matched the genome sequence imperfectly and perfectly, respectively (Table 1). A significant number of sequences from the appressoria library were shorter than 16 nt and were eliminated from further consideration. Unless stated, the remainder of the small RNA analysis refers to the data set corresponding to sequences perfectly matching the genome.

**Table 1:** Summary statistics of small RNA libraries from mycelia and appressoria tissues.

Library type	Total number of sequences	Sequences with both linkers	**Sequences**^**a**^ ≥ 16 nt	**Imperfect**^**b **^**match to genome**	**Perfect**^**c **^**match to genome**	Non-redundant **Perfect**^**d **^**match to genome**
Mycelia	318, 454	225, 381	124, 024	37, 210	32, 898	16, 745
Appressoria	343, 303	294, 864	25, 832	8, 394	6, 370	3, 315

Total	661, 757	520, 245	149, 856	77, 880	39, 268	20, 060

^a ^Total number of sequences containing both linkers and length ≥ 16 nt

^b ^Total number of sequences containing both linkers with imperfect match (80% coverage; 80% pid) and ≥ 18 nucleotides long

^c ^Total number of sequences containing both linkers with perfect match (100% coverage; 100% pid) and ≥ 16 nucleotides long

^d ^Total number of distinct sequences that perfectly match genome ≥ 16.

**Figure 1:**
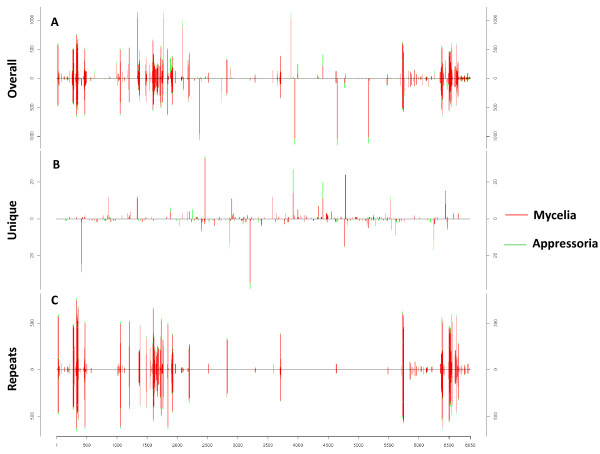
**Distribution of small RNAs with a perfect match to *M. oryzae *chromosome III**. (A) Overall distribution of small RNAs. (B) Small RNAs mapping to unique loci. (C) Small RNAs mapping to repetitive elements. Number of small RNA alignments per 5 kb of genomic sequence is shown on the Y axis with chromosome length on the X axis (vertical lines above and below chromosome line [Y = 0] represent small RNAs mapping to sense and antisense strands, respectively). Red vertical lines indicate sequences derived from mycelia and green from appressoria.

**Figure 2:**
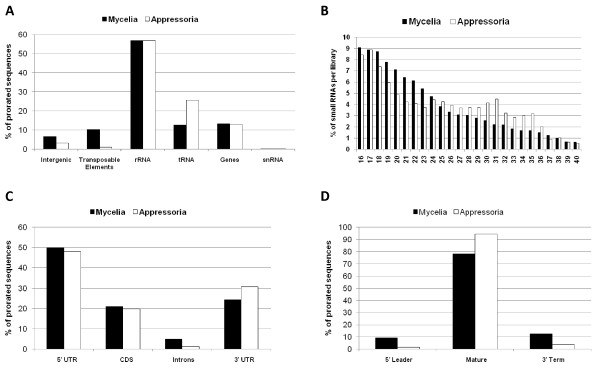
**Characterization of small RNAs from mycelia and appressoria tissues with a perfect match to the genome**. (A) Proportion of small RNAs aligned to different genomic features. (B) Size distribution of small RNAs from mycelia and appressoria libraries, (C) sub-features within genes and (D) tRNAs from mycelia and appressoria libraries.

**Figure 3:**
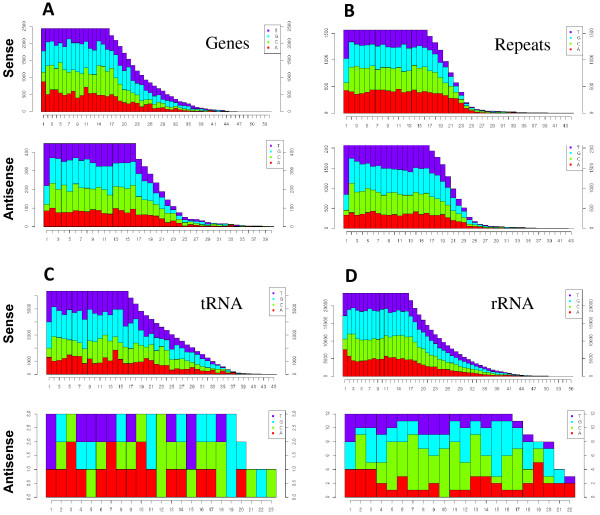
**Nucleotide composition of small RNAs mapped to *M. oryzae *genomic features**. Nucleotide frequency of small RNA alignments to sense and antisense strands of (A) genes, (B) repetitive elements, (C) tRNA and (D) rRNA loci are shown on the Y axis and nucleotide position on the X axis. Position 1 of antisense strand mapped small RNAs for genes and repeats is enriched for T (U in the RNA molecule). In contrast, C is suppressed at position 1.

Mycelia and appressoria small RNAs were mapped to the nuclear and mitochondrial genomes (Figure 1 and Additional File 1). Although small RNAs mapped throughout entire chromosomes, several genomic regions contained hundreds of alignments to both DNA strands, and corresponded mostly to repetitive DNA regions (Figure 1A). Figure 1B shows those reads that mapped only once in each library. This revealed a number of locations on each strand highly enriched for uniquely mapping reads. In contrast, many reads correspond to repetitive DNA (Figure 1C). To more thoroughly address the possible genomic feature origins of our small RNA data sets, we used prorated counts (see Material and Methods for explanation) because it takes into account multiple alignments and different features associated with each alignment (Tables 2 and 3). Overall, *M. oryzae *small RNAs aligned to a variety of genomic features including protein coding genes, tRNAs, rRNA, snRNA, repetitive elements as well as intergenic regions (Figure 2A and Tables 2 and 3). Further analysis revealed that small RNAs mapped to both the sense and antisense strand of several genomic features (Tables 2 and 3). Approximately 60% of small RNAs mapped to the sense strand of protein coding genes in both libraries, whereas small RNAs mapping to repetitive elements mapped in nearly equal frequency to both strands. In contrast, small RNAs mapping to rRNA and tRNA were highly enriched for the sense strand. Likewise, more than 90% of small RNAs originating from the mitochondria genome mapped to the sense strand. Although only a small fraction (< 3%) of small RNAs mapped to the mitochondrial genome, ~ 50% of the mitochondrial reads mapped to tRNAs and rRNA in the mycelia and appressoria, respectively. The remaining mitochondrial reads mapped to protein coding genes, rRNA and intergenic regions. Analysis of *M. oryzae *small RNA libraries also revealed a bias for U enrichment and C suppression at position 1, especially on the antisense strand for repeats and protein coding genes (CDS) (Figure 3). This result is consistent with other observations. A number of classes of small RNAs have been described with U at position 1 including 21U-RNAs in *Caenorhabditis elegans *and qiRNA, milRNAs and disiRNAs in *N. crassa *[4,14,31].

**Table 2:** Distribution of mycelia small RNAs with perfect map to nuclear and mitochondria genomes.

	**Alignment**^**a**^			**Read Count**^**b**^			**Prorated**^**c**^			**Features**^**d**^		
				
	Total	Sense	Antisense	Total	Sense	Antisense	Total	Sense	Antisense	Mapped	Total	Coverage
Genome	386741	211969	158036	32518	28592	6657	31985	26267	3569	3448	14880	23%

Genes	41318	23046	20923	8459	6213	5095	4226	2522	1704	1156	11043	10%
Introns	5396	3577	1821	1890	1848	263	209	191	17	121	19651	1%
Exons	36860	20202	19302	8270	5702	5075	4017	2331	1686	1200	30705	4%
5' UTR^e^	11416	5020	6505	4907	3147	3204	2103	971	1132	223	11241	2%
EST Supp.	3337	1715	1729	1151	502	844	691	100	590	83	2558	3%
Unsupp.	8079	3306	4777	3955	2760	2496	1412	870	542	151	8683	2%
CDS	4111	2959	1154	2463	2170	422	887	800	87	720	30486	2%
EST Supp.	2607	1636	973	1177	930	354	686	628	58	561	18154	3%
Unsupp.	1504	1324	182	1406	1302	136	201	172	29	159	12332	1%
3' UTR	23571	13686	12378	4347	3253	3342	1027	560	467	342	11076	3%
EST Supp.	3609	1882	1777	662	603	315	218	170	48	156	2551	6%
Unsupp.	19962	11805	10602	3921	2870	3243	810	390	419	198	8525	2%

tRNA^f^	30934	25184	5752	5555	5468	89	4018	3983	35	338	341	99%
5' Leader	2020	1975	47	1494	1452	44	375	375	0	112	341	33%
Mature	19615	19336	281	4243	4241	4	3137	3134	2	338	341	99%
3' Term	12937	7510	5429	1841	1799	44	506	474	32	209	341	61%
rRNA	67781	67765	18	19559	19550	11	18197	18188	9	48	48	100%
5.8 S	1630	1630	1	950	950	1	950	950	0	3	3	100%
8 S	36761	36761	1	1141	1141	1	804	804	0	41	41	100%
18 S	5185	5183	4	5185	5183	4	4242	4240	2	2	2	100%
28 S	24205	24192	15	12283	12277	8	12201	12194	7	2	2	100%

snRNA	162	162	1	129	129	1	90	90	0	8	14	57%

Transposable Elements	248528	113951	134620	3369	1522	1883	3305	1484	1821	1898	3448	55%

Intergenic	35342	-	-	4175	-	-	2150	-	-	-	-	-

Mitochondria	1448	1423	13	1445	1422	13	912	886	10	36	37	97%
Genes	961	950	13	961	950	13	216	206	10	15	15	100%
CDS	17	17	1	17	17	1	16	16	0	7	16	44%
tRNA	1074	1074	1	1073	1073	1	464	464	0	18	20	90%
Mature	346	346	1	345	345	1	107	107	0	18	20	90%
rRNA	314	314	1	314	314	1	216	216	0	2	2	100%
Intergenic	18	-	-	18	-	-	16	-	-	-	-	-

^a ^Alignment refers to the summation of small RNA alignments to any genomic feature.

^b ^Read Count represents the summation of distinct reads mapping to a given feature. Noteworthy the values for each genome feature are generally less than the sum of its sub-features due to the small RNAs mapping to multiple features (See "Material and Methods" for more details).

^c ^Prorated apportions the weight of any small RNA between alignments and features.

^d ^Features represent the proportion of genomic features mapped by small RNAs where mapped indicates the number of members for each genomic feature mapped by small RNAs among the total possible.

^e ^Values indicate number of small RNAs that mapped to sub-features within gene models that are supported by ESTs (EST Supp.) or have no EST supporting evidence (Unsupp.)

^f ^tRNA entries include pseudo-tRNAs.

**Table 3:** Distribution of appressoria small RNAs with perfect map to nuclear and mitochondria genomes.

	**Alignment**^**a**^			**Read Count**^**b**^			**Prorated**^**c**^			**Features**^**d**^		
				
	Total	Sense	Antisense	Total	Sense	Antisense	Total	Sense	Antisense	Mapped	Total	Coverage
Genome	27149	22116	6630	6352	6145	1311	6341	5768	361	1590	14880	11%

Genes	5117	2699	2846	2106	1633	1279	831	504	327	331	11043	3%
Introns	211	197	16	188	187	6	12	12	0	23	19651	0%
Exons	4965	2553	2840	2097	1545	1279	819	492	327	319	30705	1%
5' UTR^e^	1717	754	968	1220	737	678	398	226	172	70	11241	1%
EST Supp.	196	101	99	168	99	73	92	29	63	27	2558	1%
Unsupp.	1521	654	870	1057	641	606	306	197	109	45	8683	1%
CDS	307	256	53	278	255	27	165	146	19	160	30486	1%
EST Supp.	160	125	37	134	125	13	121	116	6	124	18154	1%
Unsupp.	147	132	17	147	132	17	44	31	13	36	12332	0%
3' UTR	3244	1687	1979	1193	882	928	256	119	136	101	11076	1%
EST Supp.	45	36	13	21	20	7	10	8	2	24	2551	1%
Unsupp.	3199	1652	1967	1174	865	924	246	112	134	78	8525	1%

tRNA^f^	8732	8459	275	1984	1983	3	1629	1627	2	317	341	93%
5' Leader	290	290	1	112	112	1	30	30	0	14	341	4%
Mature	8437	8437	1	1961	1961	1	1534	1534	0	177	341	52%
3' Term	957	684	275	398	397	3	65	63	2	168	341	49%

rRNA	9777	9775	4	3802	3801	3	3603	3601	2	47	48	98%
5.8 S	427	427	1	355	355	1	355	355	0	3	3	100%
8 S	3446	3446	1	99	99	1	70	70	0	41	41	100%
18 S	649	649	2	649	649	2	509	508	1	1	2	50%
28 S	5255	5254	3	2699	2699	2	2670	2669	1	2	2	100%

snRNA	7	7	1	5	5	1	3	3	0	4	14	29%

Transposable Elements	7046	3470	3578	71	34	39	63	33	30	891	3448	26%

Intergenic	1112	-	-	438	-	-	212	-	-	-	-	-

Mitochondria	39	38	2	39	38	2	29	27	1	23	37	62%
Genes	17	17	2	17	17	2	7	6	1	8	15	53%
CDS	2	2	1	2	2	1	1	1	0	2	16	13%
tRNA	17	17	1	17	17	1	8	8	0	13	20	65%
Mature	11	11	1	11	11	1	4	4	0	6	20	30%
rRNA	18	18	1	18	18	1	14	14	0	2	2	100%
Intergenic	1	-	-	1	-	-	1	-	-	-	-	-

^a ^Alignment refers to the summation of small RNA alignments to any genomic feature.

^b ^Read Count represents the summation of distinct reads mapping to a given feature. Noteworthy the values for each genome feature are generally less than the sum of its sub-features due to the small RNAs mapping to multiple features (See "Material and Methods" for more details).

^c ^Prorated apportions the weight of any small RNA between alignments and features.

^d ^Features represent the proportion of genomic features mapped by small RNAs where mapped indicates the number of members for each genomic feature mapped by small RNAs among the total possible.

^e ^Values indicate number of small RNAs that mapped to sub-features within gene models that are supported by ESTs (EST Supp.) or have no EST supporting evidence (Unsupp.).

^f ^tRNA entries include pseudo-tRNAs.

To further explore small RNA classes potentially specific, or enriched, in each tissue, we analyzed the mycelia and appressoria small RNAs in detail. First, we inspected the size distribution of small RNAs in each library type. Surprisingly, the mycelia library was enriched for small RNAs of the size 18 - 23 nt compared to 28 - 35 nt for the appressoria library (Figure 2B). Furthermore, the mycelia library was enriched for small RNAs derived from transposable elements and from intergenic regions whereas the appressoria library was enriched for small RNA originated from tRNA loci (Figure 2A). This is discussed in greater detail in the following sections. Small RNAs mapping to protein coding genes, rRNA and snRNA were present in roughly equal proportions between mycelia and appressoria libraries (Figure 2A). Regardless of tissue type, most of the small RNAs mapping to protein coding genes aligned to 5'- and 3'-UTRs (Figure 2C). Although small RNAs matched to genes at roughly equal proportions in both libraries, some genes exhibited tissue-specific mapping of small RNAs. Small RNAs originating from tRNA loci mapped predominantly to the mature tRNA sequence in both libraries (Figure 2D). Relatively few small RNAs mapped to snRNA (Figure 2A). Nevertheless, we identified several small RNAs mapping to the sense strand of different snRNAs (U2, U4 and U6) (Additional File 2). Finally, although only relatively few small RNAs mapped to mitochondrial genomic features in each library, the mycelia library contained a greater proportion of mitochondrial mappings compared to the appressoria library.

Overall, we identified small RNAs mapping to a variety of genomic features including intergenic regions. Moreover, we discovered evidence for enrichment of small RNA classes in different fungal cell types.

### Mycelia enriched small RNAs: Intergenic- and Repetitive DNA-derived small RNAs

**Figure 4:**
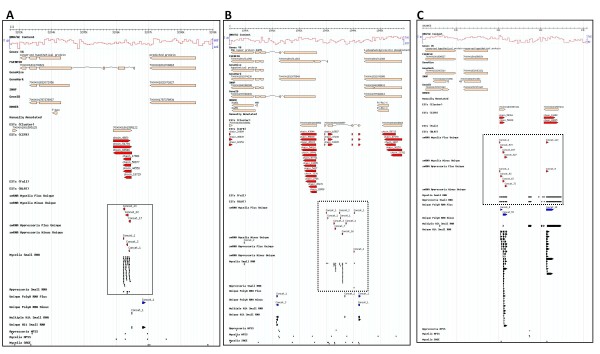
**Small RNAs mapped to intergenic features**. Small RNAs (dotted box) mapping uniquely to intergenic regions on (A) chromosome III (3202700.3209000), (B) chromosome I (1539400.1545900) and (C) unlinked DNA (9200.13100) are enriched in mycelia. ESTs and ESSs provide additional evidence for transcriptional activity within and around these intergenic regions.

Small RNAs mapping to intergenic regions were highly enriched in the mycelia compared with appressoria library. A total of 4175 (2150 prorated; 7%) small RNAs from the mycelia library mapped to intergenic regions compared to 438 (212 prorated; 3%) from the appressoria library (Figure 2A and Tables 2 and 3). To provide additional evidence for transcriptional activity for our intergenic small RNAs, we mapped these small RNAs against previous *M. oryzae *transcriptional data sets (ESTs and Expressed Short Sequence [ESS] which includes MPSS, SAGE and CPA-sRNAs). A total of 663 mycelia and 35 appressoria intergenic small RNAs overlapped with EST or ESS and to the genome sequence (Tables 4 and 5). For instance, a 300 nt region (coordinates:3206200.3206500) on chromosome III (Figure 4A) contained 60 uniquely mapped small RNAs. This region does not contain a predicted protein coding gene (*M. oryzae *genome annotation V6) but contained an intron-exon junction of a long FGENESH predicted gene model and a set of shorter ESTs. Figure 4B shows a similar intergenic example on chromosome I (coordinates:1539400.1545900), with uniquely mapped small RNAs that are supported by EST and/or ESS evidence. In other instances, we found small RNAs mapping to ESTs and precisely to the start of CPA-sRNAs (unlinked chromosome:9200.13100) (Figure 4C). Overall, we believe these data confirm the presence of previously undefined transcription units.

**Table 4:** Association of mycelia small RNAs with ESTs and ESS.

	**Genome**^**a**^		**Intergenic**^**a**^		**Genes**^**b**^	
	
	**Read Count**^**c**^	**Features**^**d**^	Read Count	Features	Read Count	Features
ESS^e^	26027	3725	480	-	6155	1637
MPSS	4174	381	73	-	1173	250
MYC	3921	237	60	-	1038	154
APP	3446	144	37	-	1071	97
SAGE	4373	268	213	-	1186	163
CPA-sRNA	25058	3076	370	-	5681	1226

EST	23918	8258	382	-	7055	6380

EST or ESS	29290	9058	663	-	8039	6847

^a ^Small RNAs that overlap the sequence of ESS and/or ESTs at the same genome or intergenic location.

^b ^Small RNAs that map to transcriptionally (ESS and/or EST) supported gene loci.

^c ^Number of small RNAs that map to a given feature and containing ESS and/or EST transcriptional evidence.

^d ^Number of features containing ESS and/or EST evidence associated with small RNAs.

^e ^ESS (Expressed Short Sequence) are either MPSS, SAGE or CPA-sRNA.

**Table 5:** Association of appressoria small RNAs with ESTs and ESS.

	**Genome**^**a**^		**Intergenic**^**a**^		**Genes**^**b**^	
	
	Read Count^c^	Features^d^	Read Count	Features	Read Count	Features
ESS^e^	5608	2249	29	-	1575	518
MPSS	900	88	5	-	401	39
MYC	817	49	4	-	325	21
APP	764	39	4	-	378	19
SAGE	1188	62	10	-	431	26
CPA-sRNA	5461	2099	20	-	1477	455

EST	4310	3436	11	-	1618	1770

EST or ESS	6018	3865	35	-	1953	2033

^a ^Small RNAs that overlap the sequence of ESS and/or ESTs at the same genome or intergenic location.

^b ^Small RNAs that map to transcriptionally (ESS and/or EST) supported gene loci.

^c ^Number of small RNAs that map to a given feature and containing ESS and/or EST transcriptional evidence.

^d ^Number of features containing ESS and/or EST evidence associated with small RNAs.

^e ^ESS (Expressed Short Sequence) are either MPSS, SAGE or CPA-sRNA.

**Figure 5:**
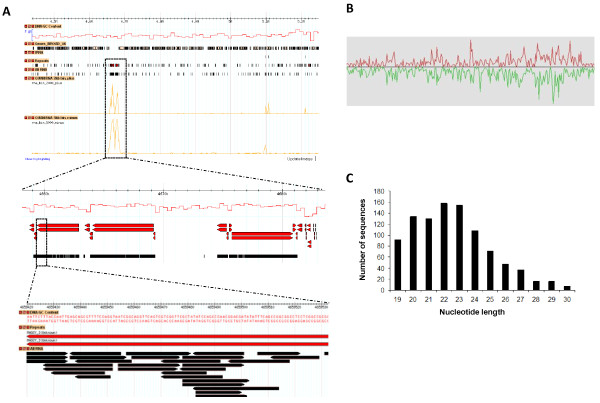
**LTR-siRNAs mapping to the retrotransposable element MAGGY**. (A) Distribution of small RNAs (black dotted box) to a ~ 0.8 Mb region of chromosome I show two major peaks on the plus (sense) and minus (antisense) strands. Closer inspection of boxed region reveals many small RNAs (black arrows) mapping to the full length (~ 5 kb) of MAGGY LTR-retrotransposon (red arrow). (B) Detailed inspection of boxed region shows LTR-siRNAs map in roughly equal proportions to both strands of MAGGY where the y axis corresponds to small RNA mappings to sense (red) and to antisense (green) strands and the X axis represents the full length MAGGY sequence. (C) Size distribution of MAGGY-derived LTR-siRNAs reveals a peak length of 22-23 nucleotides.

The mycelia library was also enriched for small RNAs that mapped to transposable elements. Approximately 10% of the *M. oryzae *genome sequence corresponds to repetitive elements [23]. In this study, we identified a total of 3369 (3305 prorated; 10%) small RNAs from the mycelia library that mapped to repetitive DNA compared to 71 (63 prorated; 1%) from the appressoria library (Figure 2A and Tables 2 and 3). In the mycelia library, 1522 (1484 prorated) small RNAs mapped to the sense strand and 1883 (1821 prorated) mapped to the antisense strand. Among the repetitive DNA classes, small RNAs mapped mainly to LTR retrotransposons and in particular to MAGGY (Figure 5 and Additional File 3). MAGGY, a well studied LTR-retrotransposon element in this fungus, contained a total of 1380 (1372 prorated) reads covering 99% of its sequence (Figure 5A and Additional File 3). MAGGY derived small RNAs mapped in roughly equal frequency to both strands (Figure 5B) and had a peak length around 22 - 23 nt long, which is characteristic of siRNAs (Figure 5C). Therefore, we propose to name *M. oryzae *small RNAs derived from LTR-retrotransposable elements as LTR-siRNAs, a sub-class of endo-siRNAs.

**Figure 6:**
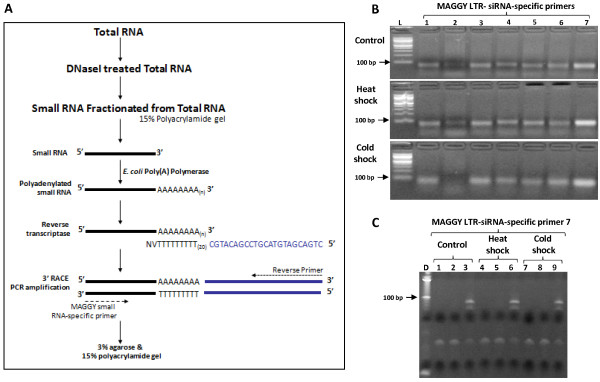
**MAGGY LTR-siRNA validation by 3' RACE**. (A) Detailed schematic flow of small RNA (< 30 nt) isolation, polyadenylation, cDNA synthesis and specific MAGGY LTR-siRNA amplification. (B) PCR amplified MAGGY LTR-siRNAs using the 3' reverse primer and 5' MAGGY small RNA-specific primer under three different mycelia growth conditions separated on a 3% agarose gel (see Material and Methods for tissue growth treatments). L = 100 bp DNA Ladder (New England Biolabs), lanes 1 - 3 and 7 correspond to small RNA mapping to sense and lanes 4 - 6 to anti-sense strands. (C) PCR amplification of MAGGY LTR-siRNA 7 on a 15% polyacrylamide gel as described in (A) and (B) above (lanes 3, 6 and 9). For PCR controls, lanes 1, 4 and 7 were loaded with water and lanes 2, 5 and 8 with total RNA. D = Decade DNA ladder (Invitrogen).

**Figure 7:**
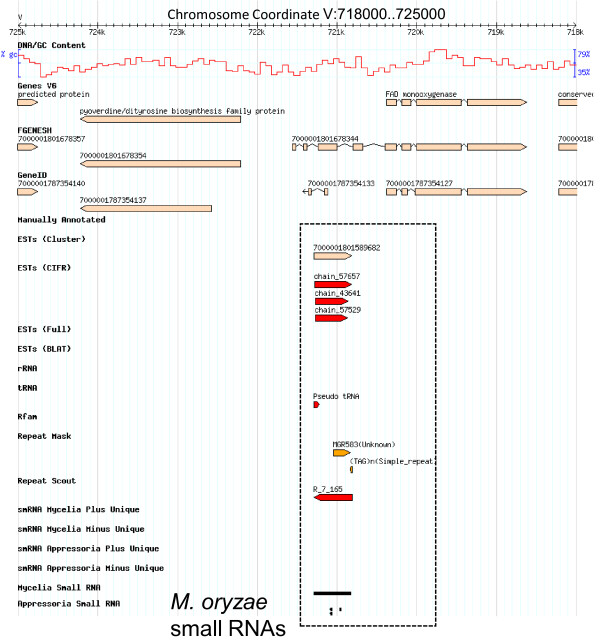
**Small RNAs mapping to MGR583**. Small RNAs mapping to the repetitive element MGR583 are highly enriched in the mycelia library (bottom of dashed box). EST data suggests MGR583 and a pseudo tRNA are transcribed as a single mRNA, which is possibly silenced by small RNAs.

In previous work, we demonstrated the value of 3' RACE PCR to validate small RNAs in *M. oryzae *(CPA-sRNAs) [25]. Here, we modified the protocol to validate MAGGY LTR-siRNAs (Figure 6A). A total of 7 MAGGY LTR-siRNAs mapping to sense and antisense strands were randomly chosen for 3'RACE PCR (Additional File 4). In addition to using RNA from mycelia grown in rich medium, we included RNA from mycelia following heat and cold shock. MAGGY LTR-siRNAs were detected at the expected sizes based on a 3% agarose gel (Figure 6B). All 7 candidates were detected across all 3 treatments except for LTR-siRNA-2 (cold shock). To further validate these findings, we repeated 3' RACE PCR for MAGGY LTR-siRNA-7, included two PCR controls (H_2_O and total RNA), and used a 15% polyacrylamide gel to gain better resolution. As expected, there was lack of amplification for both controls (lanes 1, 2, 4, 5, 7 and 8). Unexpectedly two fragments were observed for the small RNA template (lanes 3, 6 and 9) (Figure 6C). Nevertheless, one fragment corresponded to the expected size and no smear was observed as result of possible degradation. Therefore, we suggest that the second amplified fragment is potentially a modified version of MAGGY LTR-siRNA-7. Additionally, small RNAs derived from repetitive DNA also mapped to non-LTR retrotransposons such as MGR583 (Additional File 3). It is noteworthy that in many instances throughout the genome, MGR583 is surrounded by a predicted pseudo-tRNA and EST evidence suggests that they are transcribed together (Figure 7). Although it remains to be experimentally tested, our small RNA data is consistent with a role in RNA silencing to limit possible transposition of LTR and non-LTR transposable elements.

### Appressoria are enriched for tRNA-derived small RNAs

**Figure 8:**
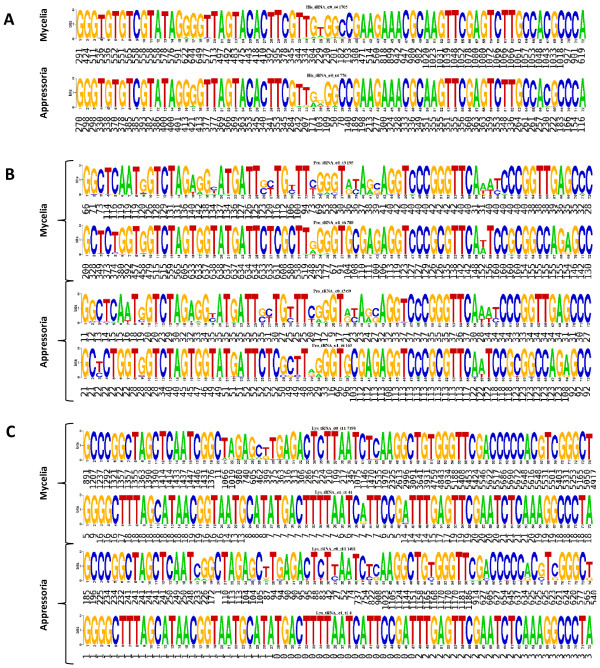
**Logos of tRFs mapping to tRNA families**. (A) Differential mapping of tRFs to the 5' and 3' halves of mature tRNAs. tRFs originating from a single tRNA^His ^type (see Material and Methods) mapped more abundantly to the 3' half in the mycelia library and in roughly equal frequency to both 5' and 3' halves in the appressoria library. Numbers below each letter in the Logos represent number of nucleotides from small RNAs mappings. (B) Members of tRNA^Pro ^family clustered into two types (two Logos per library) where small RNAs mapped predominantly to one type in both libraries. While tRFs mapped preferentially to the 5'half of both tRNA^Pro ^types in the mycelia library, they mapped primarily to the 3'half of a single type in the appressoria library. (C) Members of tRNA^Lys ^grouped into two types. In both libraries, tRFs mapped predominantly to the 3' half and preferentially to one tRNA^Lys ^type (upper Logo in each library).

**Figure 9:**
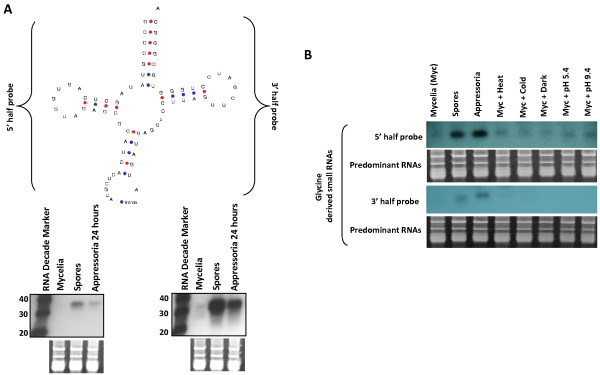
**Northern blot analysis of tRFs from different tissues and stress conditions**. (A) Northern blot hybridization analysis of tRNA^Thr ^reveals differential accumulation of 5' and 3' tRFs across different tissues. (B) 5' derived tRFs from tRNA^Gly ^accumulate under a variety of stress conditions, but most predominantly in spores and appressoria tissues. Predominant RNA signifies RNA of high quality and similar concentration.

A variety of tRNA-derived small RNAs have been reported in several organisms [9,12,32]. Here, we discovered that *M. oryzae *tRNA-derived small RNAs mapping to nuclear loci were enriched in the appressoria library (Figure 2A). A total of 5555 (4018 prorated, 13%) small RNAs from the mycelia library compared to 1984 (1629 prorated, 26%) from the appressoria library mapped to tRNA loci (Tables 2 and 3). From the mycelia library, small RNAs mapped to all 20 tRNA types (5710; 4482 prorated), Selenocysteine (SeC) (14; 13 prorated), pseudo (1034; 568 prorated) and undetermined tRNA genes (89; 2 prorated) (Additional File 5). In the appressoria library, we also observed small RNAs mapping to all tRNAs types (1989; 1636 prorated), pseudo (12; 7 prorated), and undetermined (2; 0 prorated), although none mapped to SeC (Additional File 6). Recently, Lee et al. (2009) defined tRNA-derived RNA fragments (tRFs) as small RNAs originated from a tRNA precursor which includes mature tRNA and its immediate ~ 20 nucleotides 5' and 3' flanks [12]. In this study, a small number of RNAs mapped to the 5' leader and 3' terminus of tRNAs, but most mapped to mature tRNA (Figures 2D, 8), thus we adopted this definition and named *M. oryzae *small RNAs derived from tRNA loci as tRFs. Interestingly, in many instances tRFs mapped disproportionally to either the 5' or 3' halves of mature tRNAs regardless of tissue type, although we observed an overall preference for mappings to the 3' half (Figure 8). For instance, tRFs mapped predominantly to the 3' half of tRNA^His ^in the mycelia library (Figure 8A). In contrast, tRFs mapped to both halves of tRNA^His ^in near equal frequency in the appressoria library (Figure 8A). In some instances, tRFs mapped more abundantly to the 5' half such as for tRNA^Pro ^in the mycelia tissue (Figure 8B). In other cases, such as tRNA^Lys ^and tRNA^Ala^, reads mapped predominantly to the 3' half in both libraries (Figure 8C and Additional File 7). Furthermore, we observed that some tRFs mapped preferentially to a specific tRNA type within the same tRNA family such as for tRNA^Lys ^(2 types) and tRNA^Ala ^(4 types) (Figure 8C and Additional File 7). In most cases, this maybe a reflection of the number of members of a particular tRNA type within a tRNA family. To validate tRFs, we designed primers complementary to 5' and 3' halves of tRNAs (Figure 9 and Additional File 8) and used them as probes for Northern blots. We successfully validated 5' and 3' halves of tRNA^Thr ^(MGG_20128.6) (Figure 9A) and showed that tRFs were also highly enriched in spores and present in mycelia that had been subject to various stress conditions, such as for tRNA^Gly ^(MGG_20157.6) (Figure 9B).

**Figure 10:**
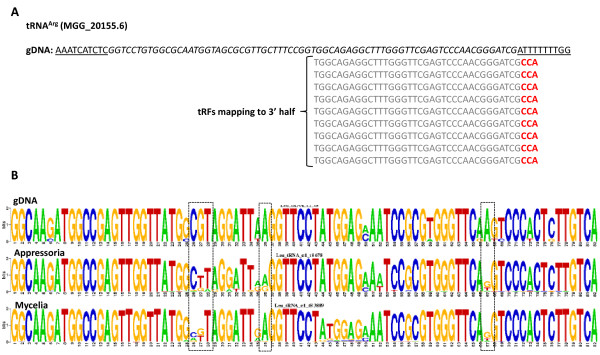
**Possible posttranscriptional modification sites of tRFs**. (A) Genomic locus of the mature tRNA^Arg ^and flanking sequence is shown in italics and underlined letters, respectively. tRFs mapping to the 3' half of tRNA^Arg ^show non-templated CCA addition (red letters). (B) Logo of genomic tRNA^Leu ^sequence (top) and corresponding tRFs Logos from appressoria (middle) and mycelia (bottom) indicate possible posttranscriptional modification sites as indicated by dotted boxes. Numbers below Logo designate nucleotide position of the mature tRNA^Leu^.

Posttranscriptional modification of tRNA has been shown to play an important role in development [33]. To explore possible posttranscriptional tRNA modification in our data sets, we analyzed a sub-set of our non-stringent small RNA data set (90% coverage and 80% sequence identity). Not surprisingly, many of these imperfectly matching tRFs were due to the presence of non-templated CCA at the 3' end, which is added during tRNA maturation [34] (Figure 10A). In other instances, small RNA reads mapping to tRNAs contained many nucleotides that did not match the genome sequences. Most likely, these typically arise during reverse transcription due to the presence of a modified base [30]. For instance, manual examination identified likely posttranscriptional modifications on tRFs nucleotides occurring at positions 26 - 28, 36 and 67 of a tRNA^Leu^, several of which have been reported previously in yeast (Figure 10B) [35]. For a comprehensive analysis, we grouped tRNA members into family types and inspected small RNA alignments for mismatches. Overall, we observed non-perfect tRFs aligning to tRNAs at a total of 56 nucleotide positions (Additional File 9). Of those, mismatches at positions 9, 25, 34 and 57 of the mature tRNA were the most frequently identified (Additional File 9). Significantly, other small RNA studies have found extensive evidence for mismatch at position 9, which suggests that posttranscriptional modification at this position is common [30]. We found numerous cases where a given nucleotide position was likely modified in both libraries. Conversely, 20 and 11 posttranscriptional modifications appeared to be specific to mycelia and appressoria, respectively (Additional File 9). Although posttranscriptional modification remains to be demonstrated as the source of our small RNA mismatched nucleotides, modification of tRNAs is known to affect stability and possibly function [32,36].

## Discussion

We have presented a detailed analysis of the small RNA transcriptome from the filamentous fungus *M. oryzae*. Because of its economic importance as a major plant pathogen of essential food staples such as rice and wheat and its molecular and classical genetic tractability, *M. oryzae *has emerged as seminal model for studying pathogen-host interactions [20,21,23,25,37]. Over the past few years, numerous genome studies have been conducted including elucidation of the genome sequence and several in depth analyses of the transcriptome. Recently, we discovered a new class of small RNAs (< 200 nt) from *M. oryzae *mycelia tissue that are methylguanosine-capped and polyadenylated (CPA-sRNAs) [25]. In an effort to further expand the repertoire of small RNAs in *M. oryzae*, we characterized small RNAs (15 - 40 nt long) from mycelia and appressoria tissues by deep 454 pyrosequencing and discovered several important differences in small RNA populations in these different tissues.

### *M. oryzae *small RNAs represent specific cleavage products

Although we can not totally exclude the possibility that some of the small RNAs were derived from random degradation, we have several lines of evidence to suggest otherwise. First, they exhibited a U enrichment at position 1. This observation has been reported in other classes of small RNAs from other eukaryotes, including fungi [4,14,31]. In *N. crassa*, it has been shown that at least three different classes of small RNAs have a strong preference for U at position 1, including qiRNAs, milRNAs and disiRNAs [4,14]. Second, our *M. oryzae *small RNAs have a preference for C suppression at position 1 which, to our knowledge, is the first time such a feature has been observed. Third, we found no correlation between small RNAs mapping to exons and gene expression (Additional File 10) [20,38]. Forth, northern blot and 3'RACE experiments provide evidence for a specific cleavage mechanism. Taken together, the presence of unique structural features, lack of correlation with gene expression and evidence for specific cleavage strongly suggest *M. oryzae *small RNAs are processed rather than products of random degradation.

### Unravelling novel intergenic small transcriptome

Small RNAs mapping to putative intergenic regions were highly enriched in vegetative mycelia compared to the appressoria library. Regardless of the precise proportion of intergenic small RNAs in *M. oryzae*, previous RNA analysis including CPA-sRNAs, MPSS, SAGE and ESTs provides compelling evidence for transcriptional activity of numerous non-coding loci (Figure 4). It is possible some of these alignments may correspond to other genomic features, particularly inaccurately annotated genes. Similar to our previous work [25], UTRs were defined as 500 bp flanking the start and stop codons of protein-coding genes in instances where full length ESTs were absent. As a consequence, it is possible that some small RNAs mapped to genes with UTRs greater than 500 bp. Alternatively, a number of predicted genes are likely inaccurately defined, or missed completely, potentially resulting in overestimation of intergenic derived small RNAs. Upon manual inspection, we identified examples where small RNAs were classified as intergenic regions but overlapped other gene predicted models; thereby satisfying the two assumptions above (Figure 4A).

In addition, many intergenic derived small RNAs corresponded to non-coding loci or possibly to other novel classes of small RNAs that remain to be discovered in *M. oryzae*. Since miRNAs are largely known to originate from intergenic regions in plants and animals, we analyzed our small RNA sequencing data with the miRDeep algorithm [39] as an approach for miRNAs discovery in *M. oryzae*. Our attempt resulted into no clear miRNA identification; although the *M. oryzae *genome has three genes coding for Argonaute proteins [23,40]. One explanation is that the miRDeep algorithm is biased to higher eukaryotes miRNAs; therefore any potential *M. oryzae *miRNA candidates slightly different from the canonical miRNAs would have been missed. Second, it is possible that they are expressed under specific stress conditions or different stages not examined here and/or are of low abundance. Finally, to our knowledge, miRNAs have not been identified in fungi and our findings are consistent with a recent review [41]. The precise identity and role these intergenic small RNAs play during growth and development in *M. oryzae *remains an important question to be addressed.

### Potential role of LTR-siRNA on genome integrity

In addition, we identified an enrichment of repeat-associated small RNAs, mapping predominantly to retrotransposable elements. Previous studies have shown that transposable elements likely play a significant role in the genome evolution of *M. oryzae *such as loss of synteny [42] and deletion of avirulence genes [43-45]. Small RNAs derived from repetitive elements have been classified as endo-siRNAs or piRNAs in plants, humans and *Drosophila *and act to silence TE elements [46-49]. In our work, small RNAs derived from repetitive elements mapped at roughly equal frequency to sense and antisense strands, which is a characteristic of the endo-siRNA-mediated RNA silencing mechanism [46,47]. We observed that most of the *M. oryzae *endo-siRNAs were derived from retrotransposable elements and were defined as LTR-siRNAs (Additional File 3). Among the TEs mapped by LTR-siRNAs, MAGGY, an extensively studied LTR-retrotransposable element found primarily in rice infecting strains, contained the highest number of small RNAs (Additional File 3). This is in contrast to Pyret, a more ancient LTR retrotransposon, which is thought to be inactive and to which few LTR-siRNAs mapped. Others have shown that MAGGY remains active. Ikeda et al (2001) demonstrated that the MAGGY promoter is activated by heat shock, copper sulfate and oxidative stress in a rice infecting strain [50]. In subsequent work, which involved introduction of MAGGY into a wheat strain (Br48), it was demonstrated that a siRNA-dependent mechanism that involves Dicer 2 was required to suppress MAGGY transposition [51]. Thus, our data are consistent with these previous findings that MAGGY is active at least to some extent. It remains to be determined whether other retrotransposable elements are suppressed by similar LTR-siRNAs based mechanisms. Although TE transposition appears to be suppressed in rice infecting strains, we suggest that *M. oryzae *cellular machinery allows limited TE movement enabling loss of avr genes, but at the same time utilizes small RNA silencing machinery to ensure genome integrity and avoid excessive deleterious mutations.

### tRNA-derived small RNAs suggest tRNA cleavage based biogenesis

In contrast to the mycelia library, our sequencing data revealed that the appressoria library had a greater accumulation of small RNAs mapping to tRNA loci (5' leader, 3' termini and mature tRNA sequence), which were named tRFs. Similar to a recent study in humans and *A. fumigatus *where small RNAs mapped to either the 5' or 3' halves of mature tRNAs and had predominant lengths of 31 - 39 nt, *M. oryzae *tRFs also mapped mostly to either 5' or 3' halves of all tRNA types and were greater than 27 nt in length [10]. In *S. cerevisiae *and human, tRNA cleavage around the anticodon loop results in small RNAs [11,13] and has been proposed as a novel mechanism for down regulation of protein synthesis [9]. In *M. oryzae*, we identified two orthologs (MGG_05342.6 and MGG_10510.6) of the yeast Rny1p, which is required for tRNA cleavage, suggesting a similar tRNA cleavage mechanism exists in *M. oryzae*.

Strikingly, tRNA cleavage is triggered by diverse stimuli such as oxidative stress, nutrient starvation, cell proliferation and development [9,12,52-54]. For instance, in the human pathogenic fungus *A. fumigatus*, tRNA cleavage is induced by conidiation and likely leads to interruption of protein synthesis by limiting availability of intact tRNA molecules [9]. In *M. oryzae*, our sequencing data and northern blots showed enrichment of tRFs in conidia and appressoria tissues compared to mycelia. As in *A. fumigatus*, *M. oryzae *conidia are resting structures and reduced metabolism is expected in order to maintain nutritional storage for conidial germination. Furthermore, conidial germination and appressorium formation in *M. oryzae *represent tightly regulated developmental stages and transcriptome analyses revealed that overall expression of genes involved in protein biosynthesis is significantly reduced [20]. Thus, it is likely that *M. oryzae *cleaves tRNAs to restrict protein synthesis during appressorium formation in order to direct cellular metabolism towards infection.

### Identification of possible tRNA posttranscriptional modifications

Recent work has shown that methylation of specific tRNA types at position C38 prevents tRNA cleavage in *Drosophila*, suggesting that posttranscriptional modifications can influence the biogenesis of tRNA-derived small RNAs [55]. In *M. oryzae*, we identified several possible posttranscriptional modifications sites. While some, such as positions 9, 26, 27, 35 agree with previous modification sites characterized in *S. cerevisiae*, the most frequent sites in *M. oryzae *tRFs occurred at positions 25 and 57. A recent study in *Arabidopsis *reported position 25 to be posttranscriptionally modified [33]. Knowledge of post-transcriptional tRNA modification in filamentous fungi is limited, thus it remains to be determined whether the sites we have discovered are common to other fungi. Our discovery of possibly tissue-specific posttranscriptional modification sites in *M. oryzae *suggests they may play an important role in development. Although this remains conjecture at this time, our findings warrant further investigation. For example, in *M. oryzae *what precisely are the tRNA modifications and what gene products are required for modifying tRNAs? Moreover, are such gene products (enzymes) essential for growth and development and what role do they play in the biogenesis of tRFs?

## Conclusions

We discovered that *M. oryzae *possesses a diverse catalog of small RNAs, which exhibited different patterns of accumulation during growth and development. However, at this time, we found no evidence for canonical miRNAs. Future studies will also focus on examining small RNAs populations in various pathogenesis mutants as well as during the infection process. Further discovery and characterization may lead to practical outcomes. For example, Tinoco et al. (2010) showed that expression of dsRNA *in planta *led to trans-specific gene silencing in fungal cells [56]. Thus, we propose that a better understanding of key small RNA players in *M. oryzae *pathogenesis-related processes may illuminate alternative strategies to engineer plants capable of modifying the *M. oryzae *small transcriptome, and suppress disease development in an effective and environmentally friendly manner.

## Methods

### Fungal strain and tissue growth conditions

Because of available genome sequence and detailed transcriptome data for *M. oryzae *strain 70-15, we used this strain to construct mycelia and appressoria small cDNA libraries. For the mycelia library, mycelia tissue were grown in liquid medium containing 0.2% yeast extract and 1.0% sucrose (YeS medium; pH 7.4) at 28°C and 200 rpm [21]. After three days, mycelia were harvested, washed with H_2_O and further used for total RNA extraction. For the appressoria library, *M. oryzae *sporulation was induced on V8 medium (10% vol./vol.) at 25°C and a 12 h light photoperiod. After ten days, spores were harvested, filtered to remove mycelia fragments through Miracloth (Calbiochem, La Jolla, CA) and the spore solution (5.0 × 10^5 ^spores/mL) was inoculated into the hydrophobic surface of GelBond Film (Rockland, ME). Sixteen hours after appressoria induction, total RNA was extracted. For validation of selected small RNAs, mycelia tissue was grown on YeS medium, as described above, and subjected to incubation for 15 minutes at 4°C and 42°C before total RNA extraction. For the dark condition and pH stress treatment, mycelia tissue was grown for three days on YeS medium in absence of light and pH adjusted to 5.4 and 9.4, respectively.

### Total RNA extraction and size fractionated small RNA isolation

Total RNA was extracted as described elsewhere [57]. Briefly, mycelia and appressoria tissues were harvested and immediately placed into a mortar containing liquid N_2 _and ground with a pestle. Powdered tissue was placed into an RNase free tube containing 20 ml of Trizol (Invitrogen). Then, 5 ml of chloroform (Sigma) was added and incubated at room temperature for 5 min. After centrifugation, the supernatant was carefully transferred into a new tube containing cold isopropanol (Fisher) and total RNA precipitated by centrifugation. The pellet was washed with 70% ethanol, dried and dissolved into 700 μl of DEPC H_2_O. Total RNA quality and quantity were checked on 1% agarose gels and on a nanodrop (Spectrophometer ND-1000-V3.3 - NanoDrop Technologies, Inc., Wilmington, DE), respectively.

To isolate small RNAs ranging from ~ 15 - 40 nt, total RNA was subjected to electrophoresis in a 15% TBE-urea polyacrylamide gel (Bio-Rad) and RNA molecules migrating between the bromophenol blue (~ 10 nt) and xylene cyanol (~ 30 nt) loading dyes were excised from the gel. Gel slices were chopped into tiny pieces and small RNAs eluted using 0.3 M NaCl, with constant agitation at 4°C over night. After centrifugation, the supernatant was placed into a new tube and extracted with phenol:chloroform:isoamyl alcohol (24:24:1) twice. Small RNAs were precipitated with 100% ethanol, 5 M-ammonium acetate (Ambion) and glycogen (Ambion). Following a 70% ethanol wash, the small RNA pellet was dried and solubilized in 50 μL of DEPC-H_2_O.

### Small RNA cDNA library and 454 sequencing

*M. oryzae *small RNA cDNA libraries were constructed according to Lu and colleagues (2007) with minor modifications [58]. Initially, size fractionated small RNAs were treated with DNaseI (New England BioLabs) to ensure the absence of any DNA contamination. Small RNA 5'-end free phosphate was removed by treatment with bacterial alkaline phosphatase (TaKaRa) at 37°C for 60 min. Next, T4 RNA ligase (Epicentre) was used to ligate 3' linkers (Additional file 11) to small RNAs. The 3' small RNA with linker was then phosphorylated with T4 polynucleotide kinase (Epicentre) and a 5' linker (Additional file 11) added by T4 RNA ligase (Epicentre). The resulting products were used as templates to synthesize cDNA using the 3' linker primer (Additional file 11) and SuperScript III (Invitrogen). RNA was hydrolyzed by RNase H (2 U/μl) (New England BioLabs). Finally, PCR was performed to amplify double-stranded cDNA using high fidelity Platinum Taq DNA polymerase (Invitrogen) and 5' and 3' linker primers (Additional file 11). cDNA from both mycelia and appressoria libraries were ligated to 454 adapters and subjected to pyrosequencing (454 Life Sciences) at the Joint Genome Institute, Walnut Creek, CA [59].

### Small RNA data analysis

To extract *M. oryzae *small RNAs reads from 454 sequencing, we identified and eliminated sequences corresponding only to 5' and 3' linkers and matched the remaining sequences to *M. oryzae *genome V6 according to the following criteria. First, a stringent BLASTN data set was identified, which consisted of matches at least 16 nt in length and having a match of 100% coverage and 100% sequence identity. Second, we considered the possibility of RNA posttranscriptional modifications and defined a non-stringent BLASTN data set having sequences ≥ 18 nt in length and imperfect match (80% coverage and 80% sequence identity). The sequence data is available at DNA Data Bank of Japan (DRZ000045 and DRZ000046).

For graphical visualization, we mapped small RNAs from both libraries throughout all *M. oryzae *chromosomes. In the first instance, every read mapping to a genomic locus was scored as an absolute value of one regardless of the number of times it matched the genome. Secondly, only unique mappings, i.e. the reads that mapped once in the *M. oryzae *genome were displayed.

For a more comprehensive analysis of our small RNAs, the same criteria we recently used for CPA-sRNAs were applied [25]. We devised this analysis to minimize genomic origin ambiguity when a small RNA aligned to several genomic loci. We used three criteria for small RNA mapping (alignments, read counts and prorating), and associated them to all nuclear and mitochondria genomic features (genes, tRNA, rRNAs, snRNAs, transposable elements and intergenic) and to other available transcriptional data (ESTs, SAGE, MPSS and CPA-sRNAs). Alignment refers to the summation of all small RNA alignments to any genomic feature. Read count represents the summation of distinct reads mapping to a given feature. For example, read (small RNA) 1 maps to tRNA^Ala ^and tRNA^Leu ^. Read 2 also maps to tRNA^Leu ^and to gene X. Then, in our analysis, the read count summary would be 2 for genome, 2 for tRNAs and 1 for genes. As read 2 mapped to more than one feature, we addressed this issue of read origin by defining prorating such that we considered any read with multiple alignments and features associated with that alignment. First, each small RNA was given a weight corresponding to the number of copies found in our sequencing libraries. Next, the weight was equally divided between all its genomic alignments. Then, given an alignment, each feature received an equal fraction of the alignment's weight. Considering the previous example, tRNA^Ala ^and tRNA^Leu ^were prorated as 0.5 each for read 1. The same applies for read 2 where tRNA^Leu ^and gene × were prorated as 0.5. In summary, tRNA feature received a prorated value of 1.5 (0.5 × 3), 0.5 for genes and 2 for genome. Last, we created sub-features such as 5' leader, mature and 3' region which received equal fractionated weight from their parent feature. Allocated small RNA weights for a given feature or sub-feature were summed to generate final prorated values.

### Posttranscriptional modification analysis

To investigate the possibility of tRNA posttranscriptional modifications, we used the non-stringent small RNA alignment data set (defined above) and used WebLogo to characterize nucleotide mismatches [60]. To conduct this analysis, for every tRNA family, we grouped tRNA genomic sequences by tRNA type (80% sequence identity) and created a consensus logo. For example, the *M. oryzae *genome contains 7 tRNAs for Asn, which grouped into 3 distinct types. Second, all small RNA reads in the appressoria or mycelia library or combined that aligned to a given tRNA cluster (80% coverage and 90% sequence identity) were used to generate logos. Finally, the number of reads per nucleotide position used to create the Logos was determined. To identify possible posttranscriptional modification sites, two rules were imposed. First, nucleotide positions in the genomic reference logo that were polymorphic were excluded for further consideration. Second, only nucleotide positions in small RNA logos which showed variant nucleotides with a bit score ≤ 1 were considered.

### Northern blot and 3' RACE analysis of small RNAs

Mycelia and appressoria total RNA were extracted as described previously. Total RNA was also extracted from 10 days old spores. For northern blots, 10 μg of total RNA per tissue were loaded into a 15% TBE-urea polyacrylamide gel (BioRad). After electrophoresis (153 V for 60 min), total RNA was transferred overnight by capillary action to hybond N membrane (Amersham). The UV cross-linked membrane was prehybridized with PerfectHyb™ plus hybridization buffer (Sigma) for 30 min at 40°C. Alternatively, a homemade buffer was used. Oligonucleotide probes specific to mature tRNAs (Additional file 8) were end labeled with [γ-^32^P] ATP and hybridized overnight at the same temperature. Membranes were washed twice at 42°C using with 2 × SSC, 0.1% SDS for 10 min. before being exposed to autoradiographic film (Denville Scientific) at - 80°C. Images were developed using a Medical Film Processor (Model SRX-101A) and the detected fragments compared to the RNA Decade™ marker (Ambion). For 3' RACE experiments, size fractionated small RNAs were polyadenylated using a Poly(A) Tailing Kit (Ambion) and cDNA synthesized using SuperScript III (Invitrogen) and a 3'-oligo(dT)_20_VN linker (Additional file 4). To validate MAGGY LTR-siRNAs, a reverse primer specific for the 3'-oligo (dT)_20_VN linker was used in combination with distinct forward primers specific to MAGGY-derived small RNAs (Additional file 4) and subjected to PCR amplification. A 3% agarose gel was used to resolve PCR products (130 V; 20 min).

## Authors' contributions

CCN carried out the experiments, participated in the data analysis and drafted the manuscript. CCN, MG, TKM and RAD designed the experiments. JS, MX, DB and YYO carried out bioinformatics analysis. FC sequenced small RNA libraries. RAD participated in the coordination of experiments, data analysis, and edited the manuscript. All authors read and approved the final manuscript.

## Supplementary Material

Additional file 1 **Distribution of small RNAs with a perfect match to *M. oryzae *nuclear and mitochondrial genomes and unlinked chromosome**. (A) Overall distribution of small RNAs. (B) Small RNAs mapping to unique loci. (C) Small RNAs mapping to repetitive elements. Number of small RNA alignments per 5 kb of genomic sequence is shown on the Y axis with chromosome length on the X axis (vertical lines above and below chromosome line [Y = 0] represent small RNAs mapping to sense and antisense strands, respectively). Red vertical lines indicate sequences derived from mycelia and green from appressoria. Click here for file

Additional file 2 **Small RNAs with perfect match to snRNAs**. snRNA-derived small RNAs mapped predominantly to 3' end of U2 (A) and U4 (B). In contrast, U6-derivd small RNA mapped largely toward to the 5' end (C). Click here for file

Additional file 3 **Distribution of mycelia small RNAs mapped to repetitive elements**. Small RNAs originated from repetitive elements mapped primarily to the LTR retrotransposable element class (grey highlight) including MAGGY. Click here for file

Additional file 4 **Primers and linker used for 3' RACE**. A total of 7 distinct 5' primers corresponding to the LTR retrotransposable element MAGGY were paired with a 3' RACE primer to independently validate sense and antisense MAGGY-derived LTR-siRNA. Click here for file

Additional file 5 **Distribution of mycelia small RNAs mapped to tRNAs**. Click here for file

Additional file 6 **Distribution of appressoria small RNAs mapped to tRNAs**. Click here for file

Additional file 7 **Logos of tRFs mapping to tRNA^Ala^**. Members of tRNA^Ala ^grouped into four types. In both libraries, tRFs mapped predominantly to the 3' half and preferentially to one tRNA^Ala ^type. Click here for file

Additional file 8 **End labelled oligonucleotides used for Northern blot**. Click here for file

Additional file 9 **Distribution of tRF posttranscriptional modification sites**. Click here for file

Additional file 10 **Correlation analysis of *M. oryzae *gene expression and number of mapped small RNAs**. (A) Mycelia: gene expression (Y axis, NCBI GEO Accession GSE2716) in fungal tissue grown for 48 h (minimum medium) compared to the prorated values of small RNAs (X axis) isolated from fungus grown for 48 h in YeS medium. (B) Appressoria: gene expression data (12 h, GSE1945) compared to prorated values of small RNAs (16 h) from spores induced to form appressoria on a hydrophobic surface. Click here for file

Additional file 11 **Linkers and primers used for small RNA library preparation**. Click here for file
