# Supplementary material for: Diverse and tissue-enriched small RNAs in the plant pathogenic fungus, Magnaporthe oryzae
Source: BMC Genomics. 2011 Jun 2;12:288. doi: 10.1186/1471-2164-12-288 (PMC3132168; doi:10.1186/1471-2164-12-288)
Supplement: Additional file 1 — Distribution of small RNAs with a perfect match to M. oryzae nuclear and mitochondrial genomes and unlinked chromosome. (A) Overall distribution of small RNAs. (B) Small RNAs mapping to unique loci. (C) Small RNAs mapping to repetitive elements. Number of small RNA alignments per 5 kb of genomic sequence is shown on the Y axis with chromosome length on the X axis (vertical lines above and below chromosome line [Y = 0] represent small RNAs mapping to sense and antisense strands, respectively). Red vertical lines indicate sequences derived from mycelia and green from appressoria. [file 1471-2164-12-288-S1.PPTX]

## Slide 1
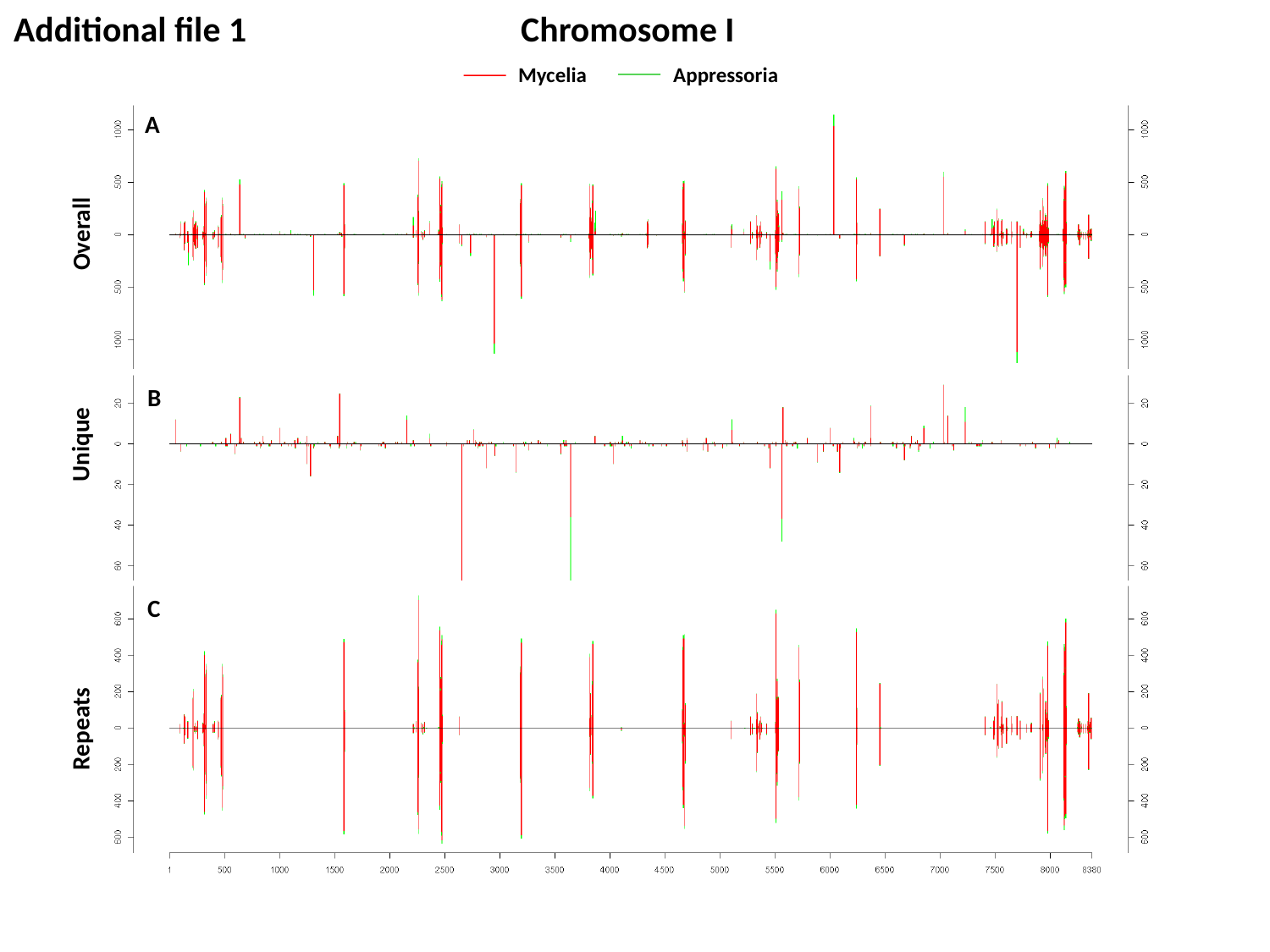

Additional file 1
Chromosome I
Mycelia
Appressoria
A
Overall
B
Unique
C
Repeats

## Slide 2
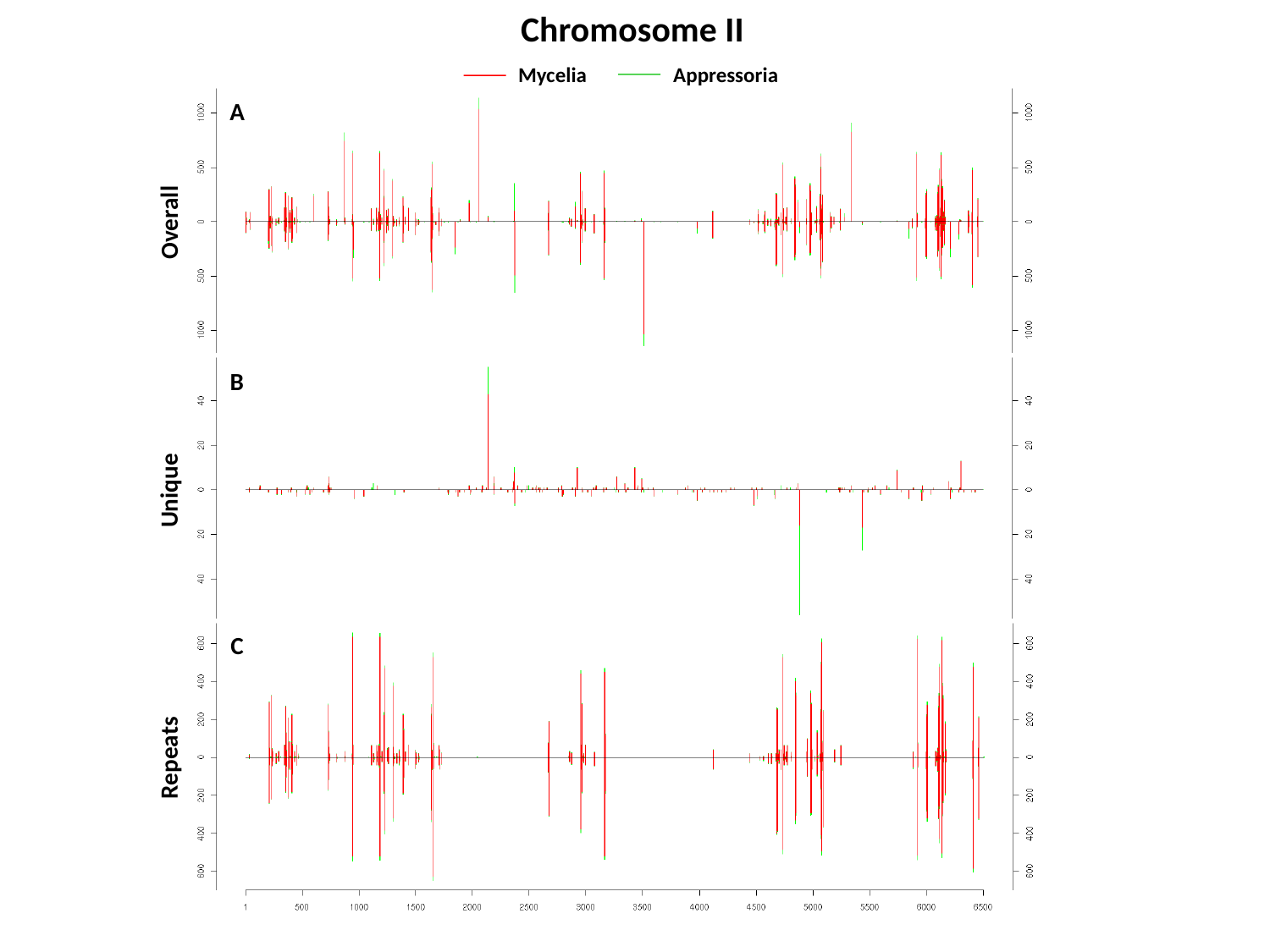

Chromosome II
Mycelia
Appressoria
A
Overall
B
Unique
C
Repeats

## Slide 3
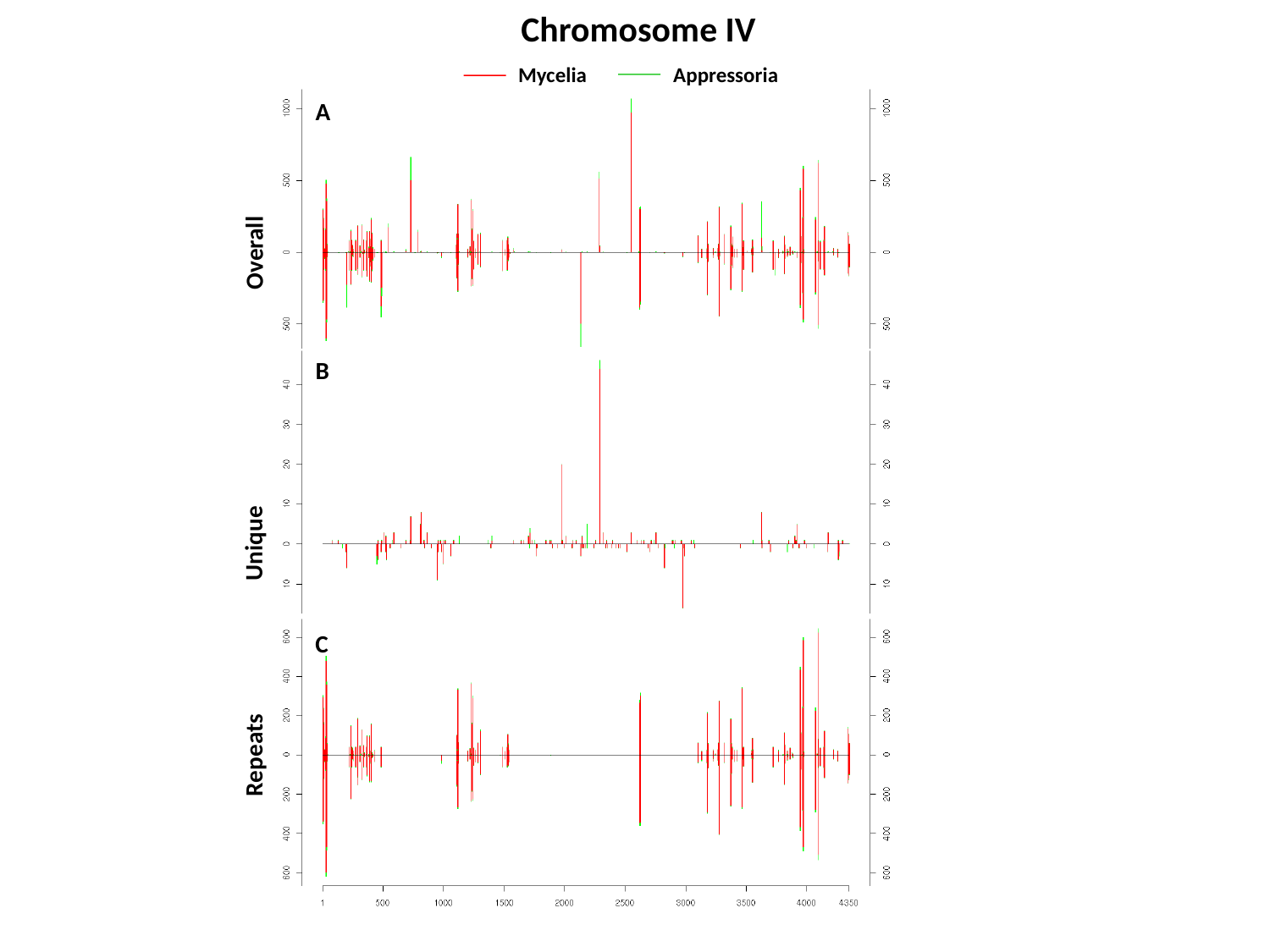

Chromosome IV
Mycelia
Appressoria
A
Overall
B
Unique
C
Repeats

## Slide 4
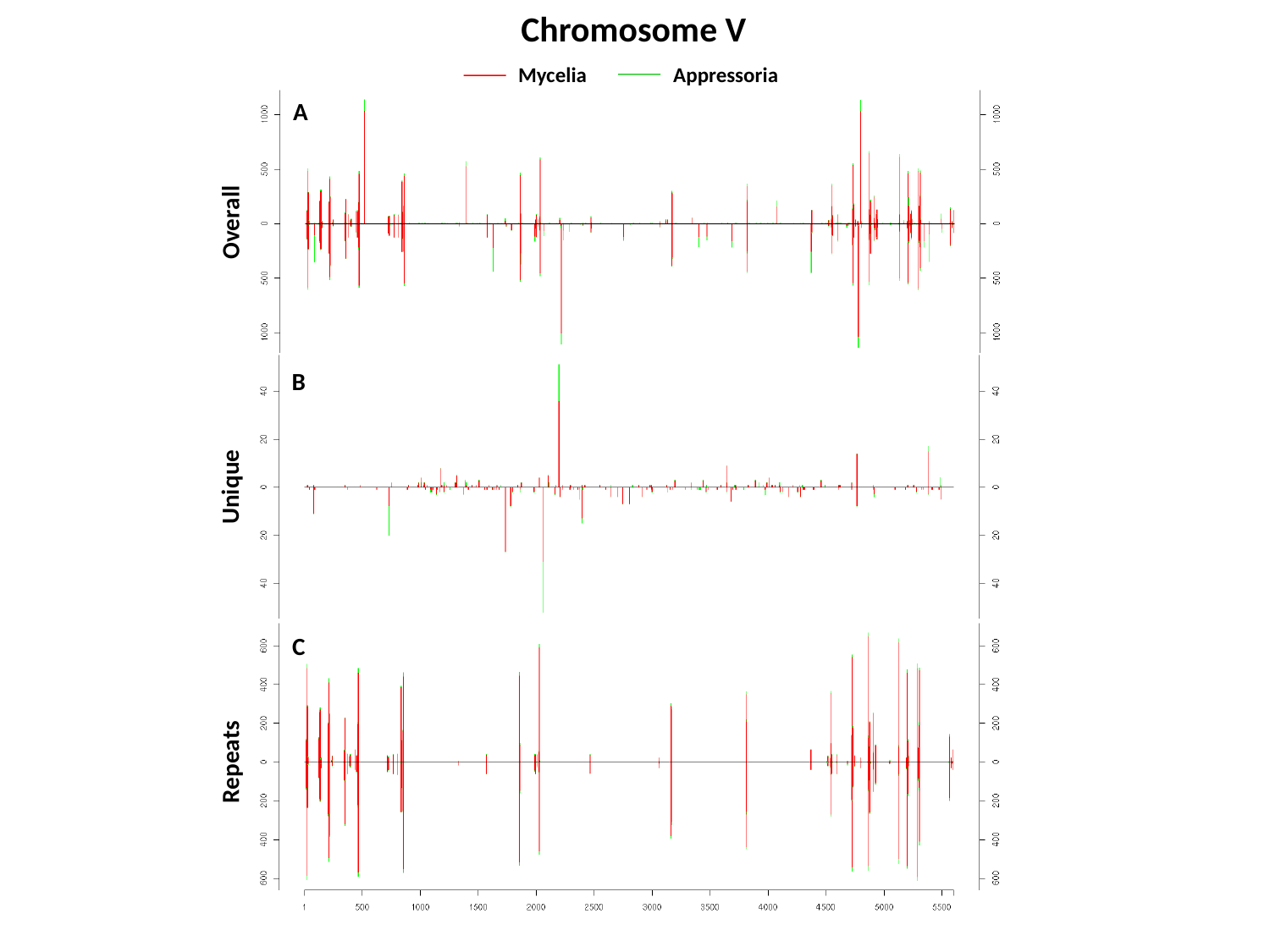

Chromosome V
Mycelia
Appressoria
A
Overall
B
Unique
C
Repeats

## Slide 5
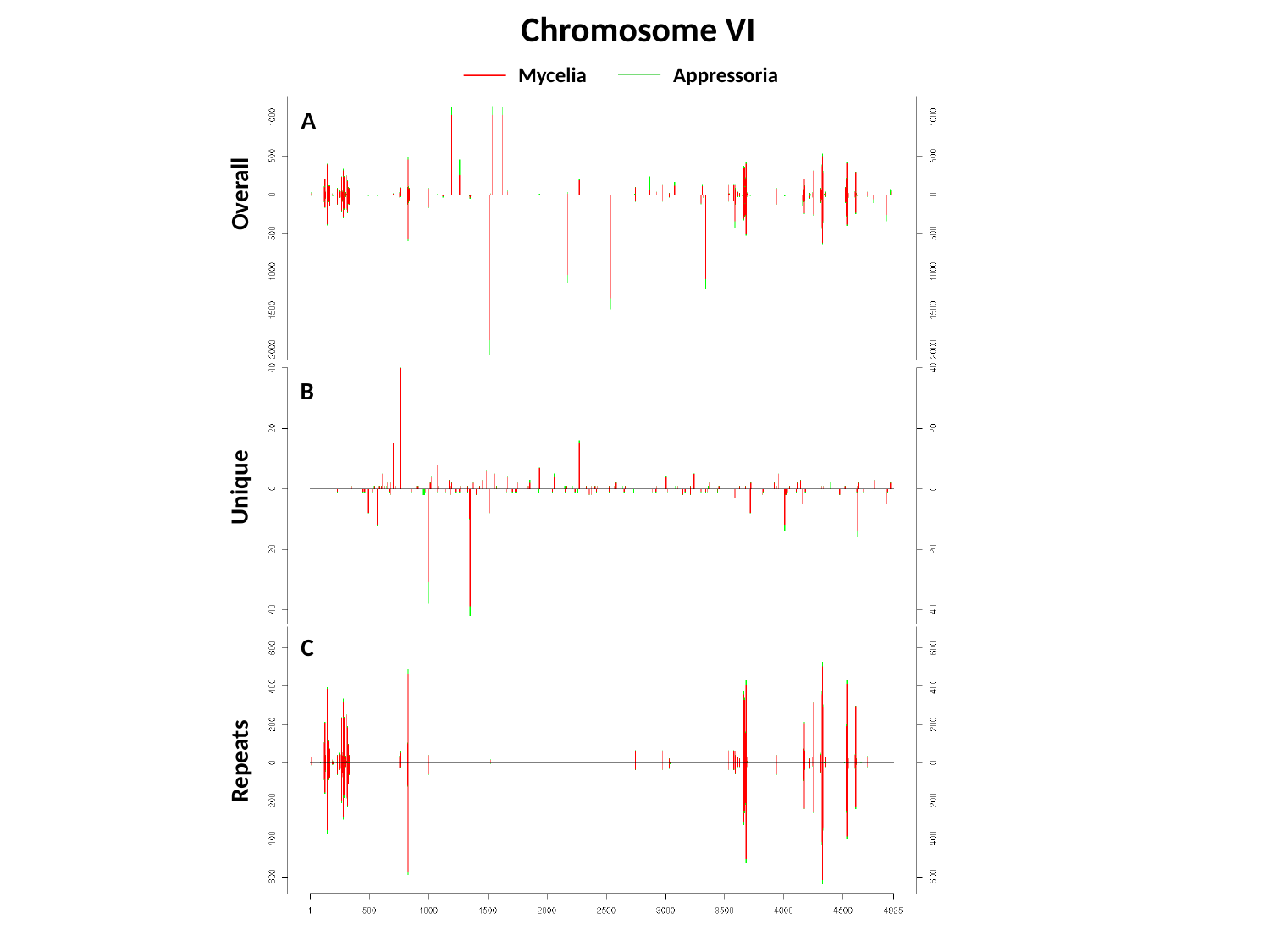

Chromosome VI
Mycelia
Appressoria
A
Overall
B
Unique
C
Repeats

## Slide 6
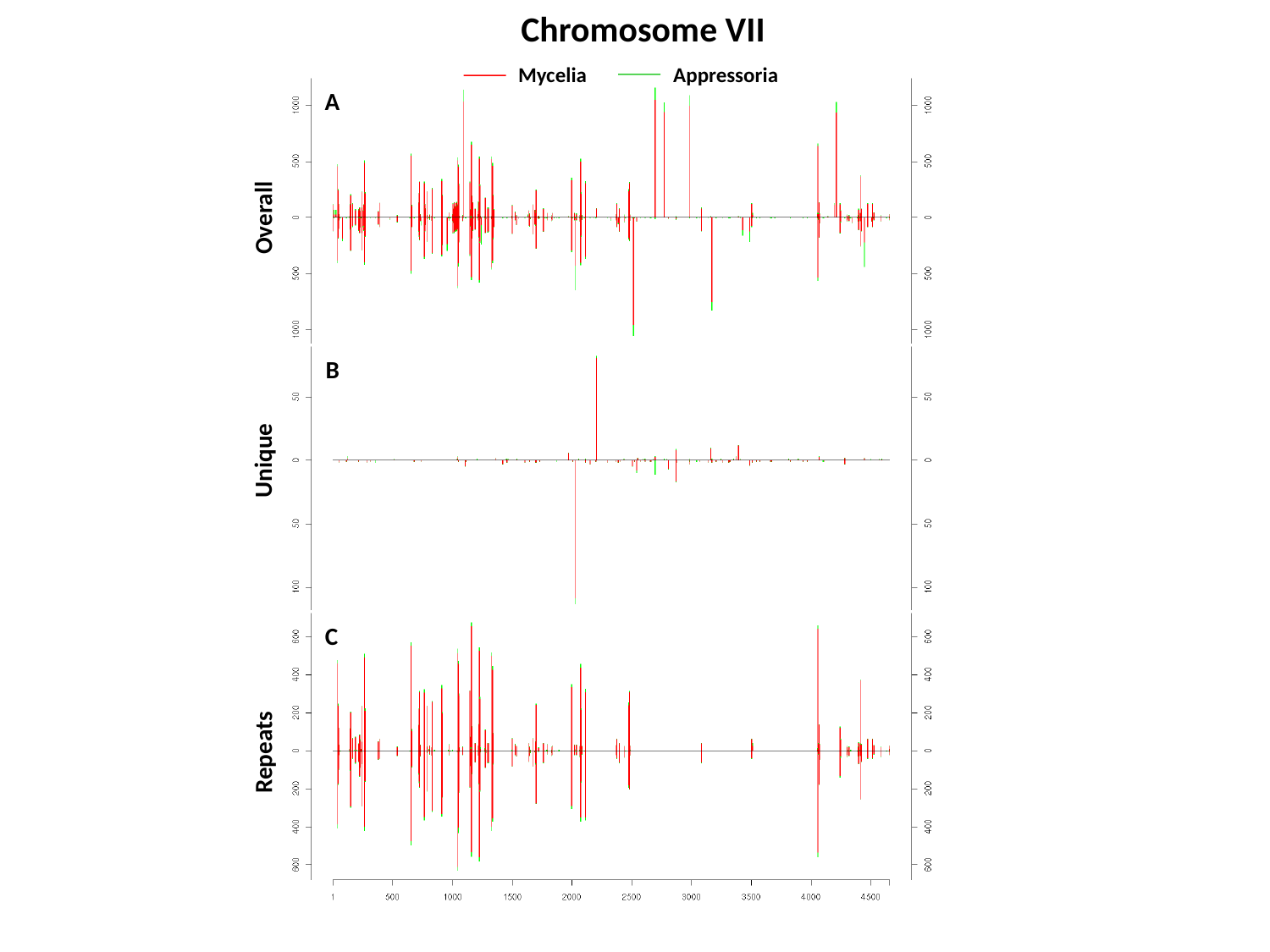

Chromosome VII
Mycelia
Appressoria
A
Overall
B
Unique
C
Repeats

## Slide 7
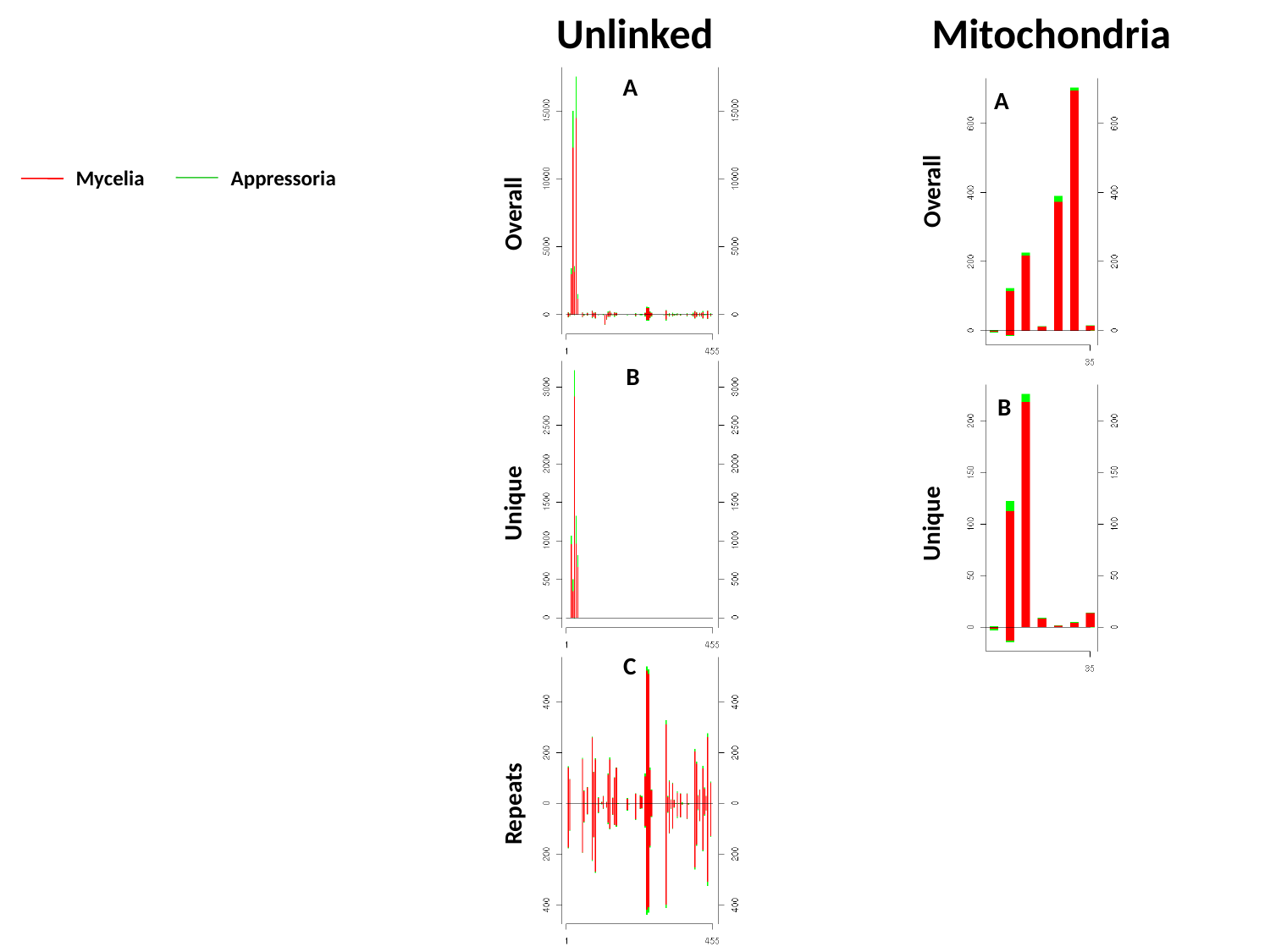

Unlinked
Mitochondria
A
A
Mycelia
Appressoria
Overall
Overall
B
B
Unique
Unique
C
Repeats
