# Supplementary figures and images for: Diverse and tissue-enriched small RNAs in the plant pathogenic fungus, Magnaporthe oryzae
Source: BMC Genomics. 2011 Jun 2;12:288. doi: 10.1186/1471-2164-12-288 (PMC3132168; doi:10.1186/1471-2164-12-288)

## Slide 1
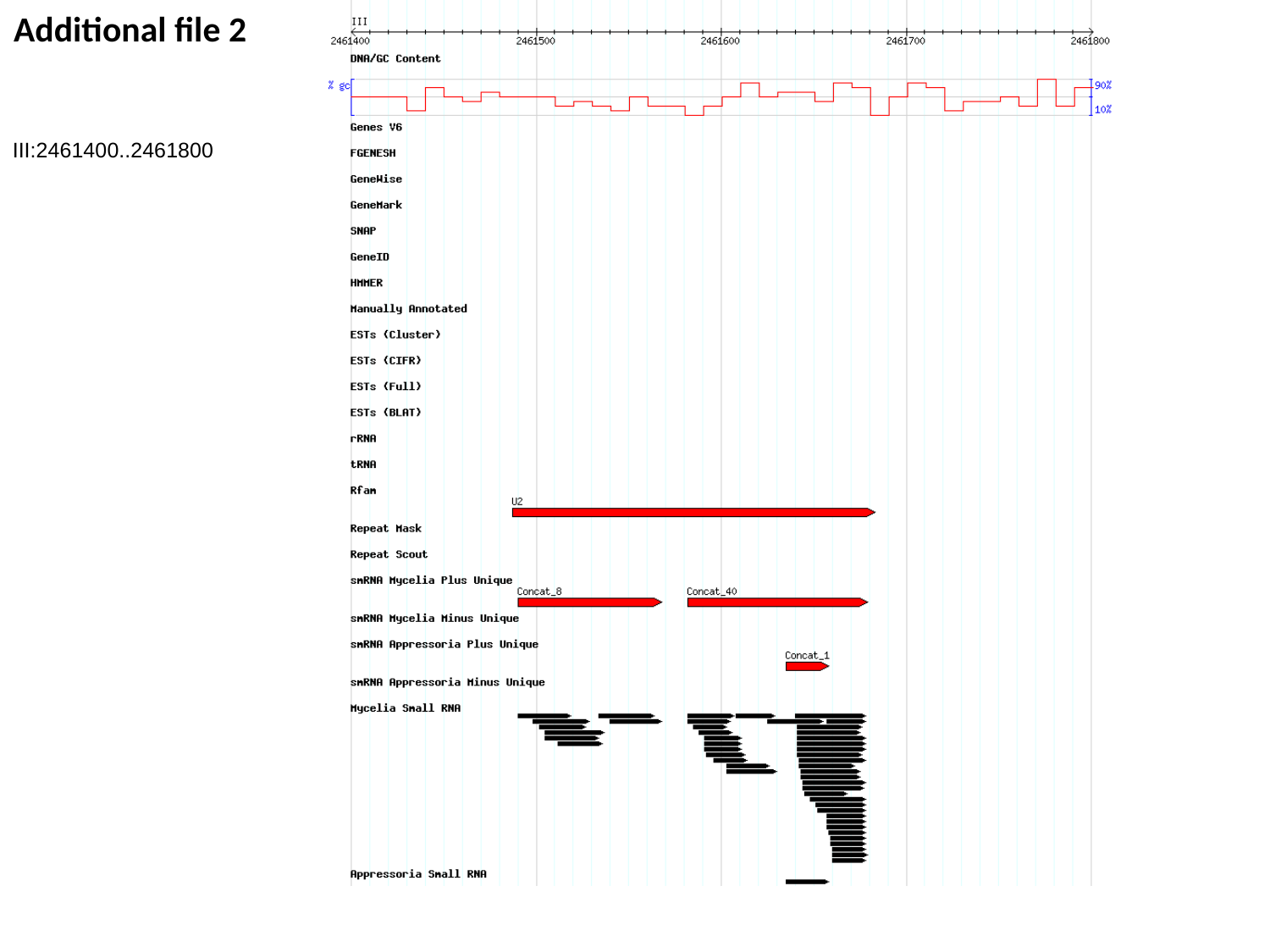

Additional file 2
III:2461400..2461800

## Slide 2
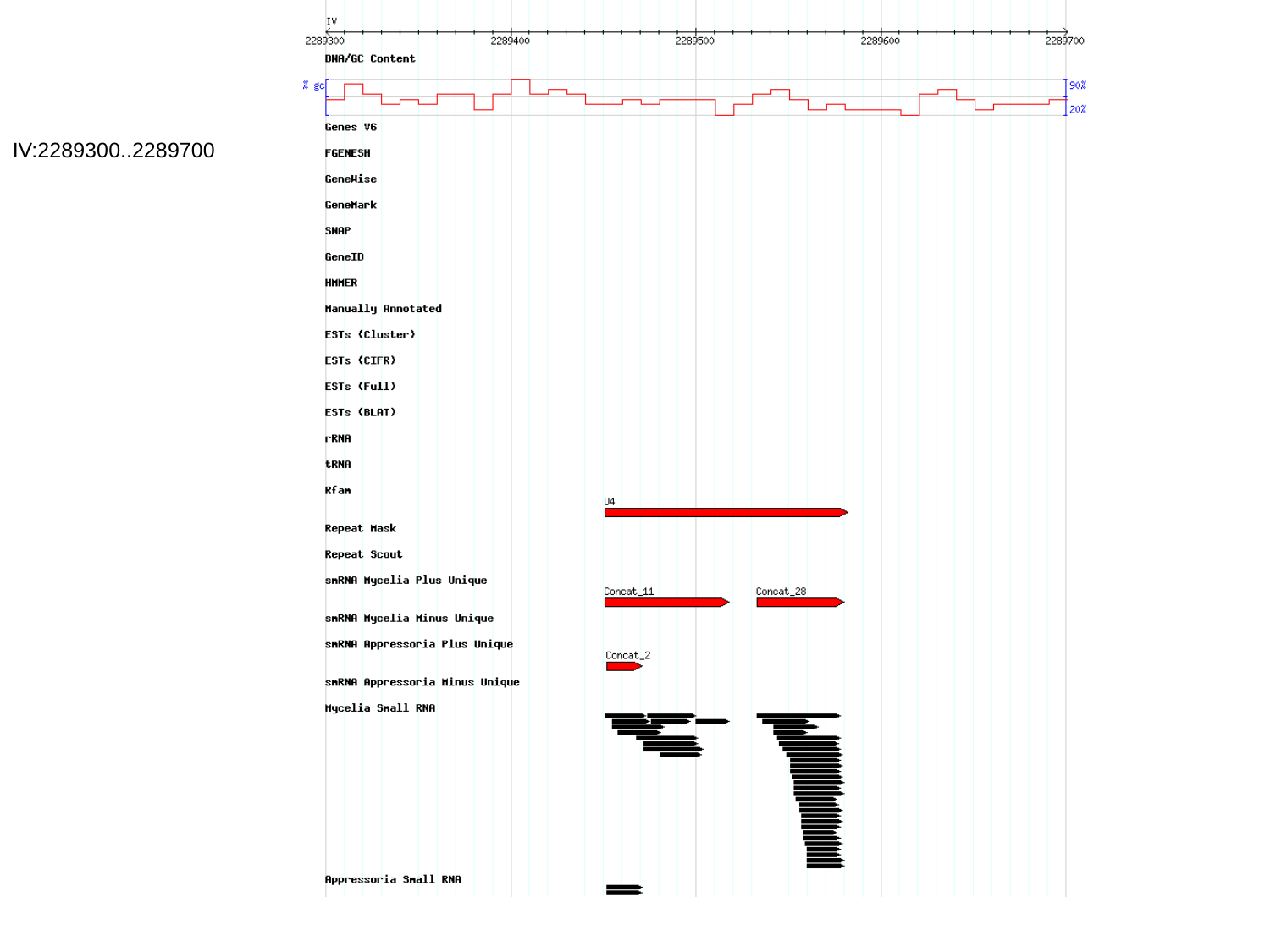

IV:2289300..2289700

## Slide 3
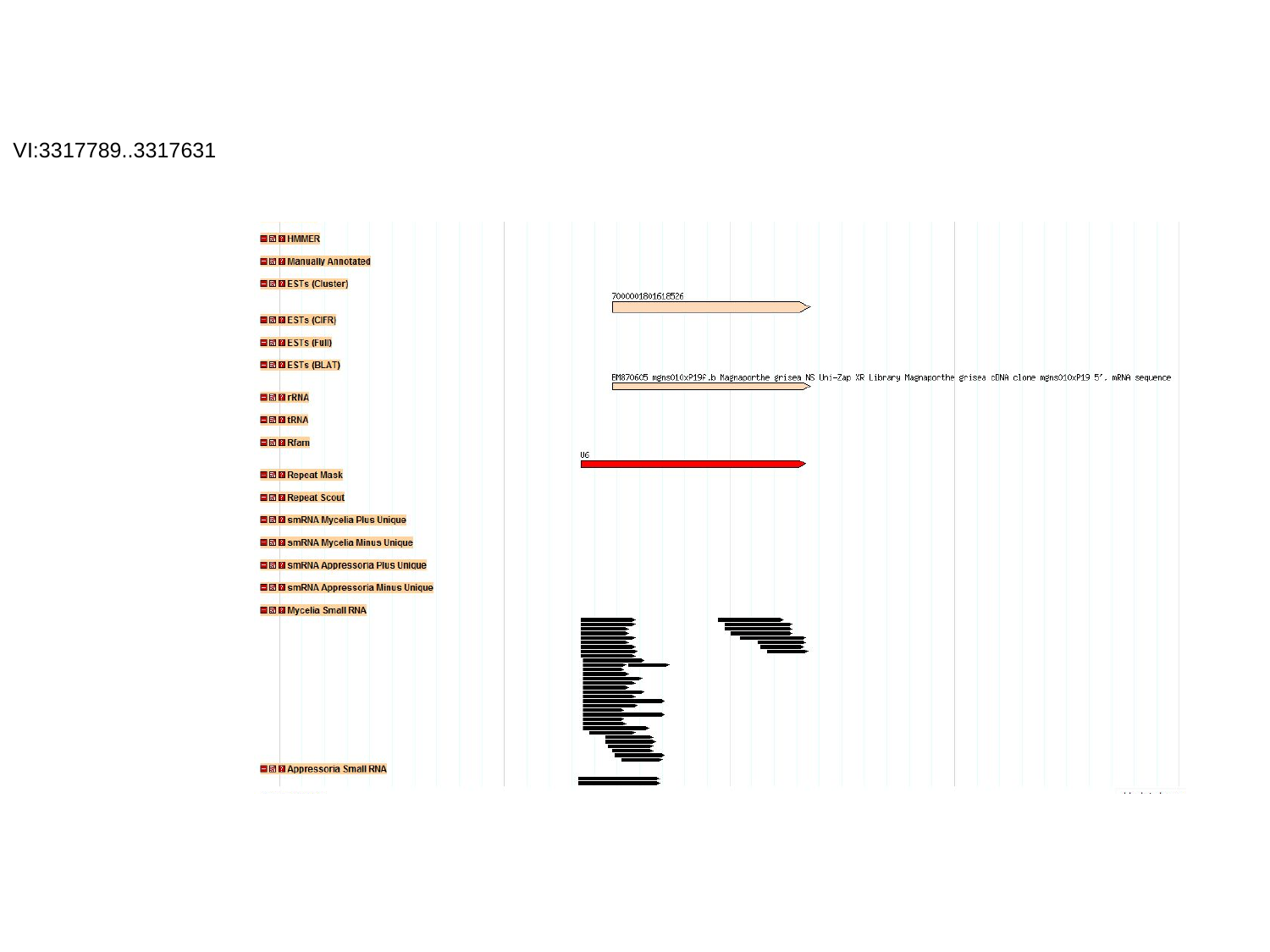

VI:3317789..3317631

Supplement: Additional file 2 — Small RNAs with perfect match to snRNAs. snRNA-derived small RNAs mapped predominantly to 3' end of U2 (A) and U4 (B). In contrast, U6-derivd small RNA mapped largely toward to the 5' end (C). [file 1471-2164-12-288-S2.PPTX]

## Slide 1
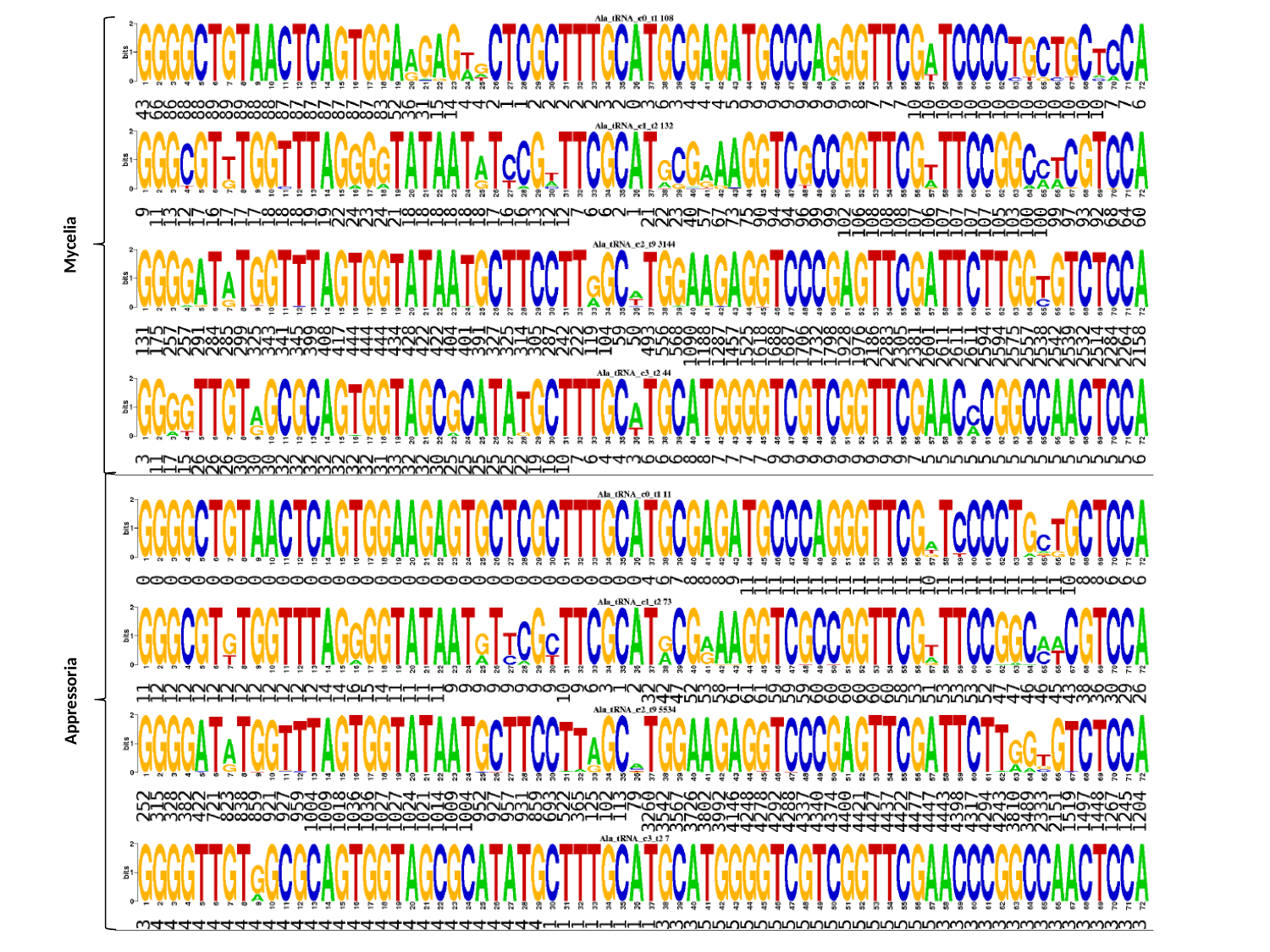

Mycelia
Appressoria

Supplement: Additional file 7 — Logos of tRFs mapping to tRNAAla. Members of tRNAAla grouped into four types. In both libraries, tRFs mapped predominantly to the 3' half and preferentially to one tRNAAla type. [file 1471-2164-12-288-S7.PPTX]
