# Supplementary material for: Diverse and tissue-enriched small RNAs in the plant pathogenic fungus, Magnaporthe oryzae
Source: BMC Genomics. 2011 Jun 2;12:288. doi: 10.1186/1471-2164-12-288 (PMC3132168; doi:10.1186/1471-2164-12-288)
Supplement: Additional file 3 — Distribution of mycelia small RNAs mapped to repetitive elements. Small RNAs originated from repetitive elements mapped primarily to the LTR retrotransposable element class (grey highlight) including MAGGY. [file 1471-2164-12-288-S3.DOCX]

**Additional file 3** – Distribution of mycelia small RNAs mapped to repetitive elements

|  | Alignment^a^ | | |  | Read Count^b^ | | |  | Prorated^c^ | | |  | Features^d^ | | |
| --- | --- | --- | --- | --- | --- | --- | --- | --- | --- | --- | --- | --- | --- | --- | --- |
|  | Total | Sense | Antisense |  | Total | Sense | Antisense |  | Total | Sense | Antisense |  | Mapped | Total | Coverage |
| Transposable Elements | 248528 | 113951 | 134620 |  | 3369 | 1522 | 1883 |  | 3305 | 1484 | 1821 |  | 1898 | 3448 | 55% |
| AFUT1 | 0 | 0 | 0 |  | 0 | 0 | 0 |  | 0 | 0 | 0 |  | 0 | 1 | 0% |
| BCBOTYPOL | 0 | 0 | 0 |  | 0 | 0 | 0 |  | 0 | 0 | 0 |  | 0 | 36 | 0% |
| GYMAG1_I | 25380 | 10370 | 15012 |  | 955 | 408 | 549 |  | 953 | 405 | 548 |  | 72 | 78 | 92% |
| GYMAG1_LTR | 499 | 152 | 349 |  | 4 | 2 | 4 |  | 4 | 1 | 3 |  | 250 | 303 | 83% |
| GYMAG2_I | 21639 | 9684 | 11957 |  | 742 | 331 | 413 |  | 739 | 329 | 410 |  | 75 | 81 | 93% |
| GYMAG2_LTR | 409 | 139 | 272 |  | 12 | 3 | 11 |  | 7 | 2 | 5 |  | 118 | 155 | 76% |
| Gypsy-1-I_AN | 0 | 0 | 0 |  | 0 | 0 | 0 |  | 0 | 0 | 0 |  | 0 | 2 | 0% |
| GYPSY1_MG | 0 | 0 | 0 |  | 0 | 0 | 0 |  | 0 | 0 | 0 |  | 0 | 10 | 0% |
| Gypsy2-I_AO | 0 | 0 | 0 |  | 0 | 0 | 0 |  | 0 | 0 | 0 |  | 0 | 3 | 0% |
| Helitron-1_AN | 0 | 0 | 0 |  | 0 | 0 | 0 |  | 0 | 0 | 0 |  | 0 | 1 | 0% |
| Hop | 0 | 0 | 0 |  | 0 | 0 | 0 |  | 0 | 0 | 0 |  | 0 | 3 | 0% |
| MAGGY_I | 167086 | 75618 | 91470 |  | 1353 | 620 | 735 |  | 1347 | 616 | 731 |  | 326 | 329 | 99% |
| MAGGY_LTR | 7282 | 2250 | 5034 |  | 27 | 10 | 19 |  | 25 | 8 | 17 |  | 360 | 365 | 99% |
| Mariner-1_AF | 0 | 0 | 0 |  | 0 | 0 | 0 |  | 0 | 0 | 0 |  | 0 | 1 | 0% |
| Mariner-6_AN | 0 | 0 | 0 |  | 0 | 0 | 0 |  | 0 | 0 | 0 |  | 0 | 1 | 0% |
| MARY1_TM | 0 | 0 | 0 |  | 0 | 0 | 0 |  | 0 | 0 | 0 |  | 0 | 1 | 0% |
| MGR583 | 24865 | 14988 | 9879 |  | 157 | 95 | 67 |  | 111 | 72 | 39 |  | 388 | 425 | 91% |
| MGRL3_I | 821 | 350 | 473 |  | 96 | 43 | 55 |  | 94 | 42 | 52 |  | 24 | 39 | 62% |
| MGRL3_LTR | 1 | 1 | 1 |  | 1 | 1 | 1 |  | 0 | 0 | 0 |  | 1 | 93 | 1% |
| MOLLY_SN | 119 | 119 | 1 |  | 3 | 3 | 1 |  | 2 | 2 | 0 |  | 60 | 70 | 86% |
| NHT2_I | 0 | 0 | 0 |  | 0 | 0 | 0 |  | 0 | 0 | 0 |  | 0 | 1 | 0% |
| OCCAN_MG | 12 | 8 | 6 |  | 12 | 8 | 6 |  | 0 | 0 | 0 |  | 2 | 97 | 2% |
| POT2 | 162 | 146 | 18 |  | 28 | 13 | 17 |  | 3 | 2 | 1 |  | 137 | 414 | 33% |
| PYRET_I | 139 | 111 | 30 |  | 16 | 14 | 8 |  | 7 | 3 | 3 |  | 67 | 499 | 13% |
| PYRET_LTR | 4 | 4 | 1 |  | 2 | 2 | 1 |  | 0 | 0 | 0 |  | 4 | 417 | 1% |
| REALAA_I | 0 | 0 | 0 |  | 0 | 0 | 0 |  | 0 | 0 | 0 |  | 0 | 2 | 0% |
| SKIPPY | 0 | 0 | 0 |  | 0 | 0 | 0 |  | 0 | 0 | 0 |  | 0 | 6 | 0% |
| TCN1-I | 0 | 0 | 0 |  | 0 | 0 | 0 |  | 0 | 0 | 0 |  | 0 | 1 | 0% |
| TY3 | 56 | 1 | 56 |  | 29 | 1 | 29 |  | 12 | 0 | 12 |  | 2 | 2 | 100% |
| U35230 | 546 | 314 | 234 |  | 92 | 57 | 37 |  | 2 | 1 | 1 |  | 12 | 12 | 100% |

^a^ Alignment refers to the summation of small RNA alignments to any genomic feature.

^b^ Read Count represents the summation of distinct reads mapping to a given feature. Noteworthy the values for each genome feature are generally less than the sum of its sub-features due to the small RNAs mapping to multiple features (See “Material and Methods” for more details).

^c^ Prorated apportions the weight of any small RNA between alignments and features.

^d^ Features represent the proportion of genomic features mapped by small RNAs where mapped indicates the number of members for each genomic feature mapped by small RNAs among the total possible.
