# Supplementary material for: Diverse and tissue-enriched small RNAs in the plant pathogenic fungus, Magnaporthe oryzae
Source: BMC Genomics. 2011 Jun 2;12:288. doi: 10.1186/1471-2164-12-288 (PMC3132168; doi:10.1186/1471-2164-12-288)
Supplement: Additional file 4 — Primers and linker used for 3' RACE. A total of 7 distinct 5' primers corresponding to the LTR retrotransposable element MAGGY were paired with a 3' RACE primer to independently validate sense and antisense MAGGY-derived LTR-siRNA. [file 1471-2164-12-288-S4.DOCX]

**Additional file 4** – Primers and linker used for in 3’ RACE.

| Primer ID | Sequence | Length (bp) | Oligo(dT)_20_ VN length^a^ | Expected fragment |
| --- | --- | --- | --- | --- |
| MAGGY LTR-siRNA 1 | AAGCCCCCGTACTGGCGCACTT | 22 | 45 | 67 |
| MAGGY LTR-siRNA 2 | AATTGCTAGCCATTATCCGCTGTTTTGAAG | 30 | 45 | 75 |
| MAGGY LTR-siRNA 3 | CAGAGGATATGCCTGAGGAGGG | 22 | 45 | 67 |
| MAGGY LTR-siRNA 4 | ATAGGGGTTTGTTTTGGCGGATTATGGGTT | 30 | 45 | 75 |
| MAGGY LTR-siRNA 5 | AAATCTTGACAGGAATTCGGCCC | 23 | 45 | 68 |
| MAGGY LTR-siRNA 6 | CGTAAATCTCGTAATTGCATTCGG | 24 | 45 | 69 |
| MAGGY LTR-siRNA 7 | GCCCAGTGGCTGTCCTTCAGGTCT | 24 | 45 | 69 |

^a^ **Oligo(dT)20VN 3’-RACE linker sequence**: CTGACGATGTACGTCCGACATGC(T)_20_VN, where V means A/G/C and N means any nt.

**3’-RACE primer:** CTGACGATGTACGTCCGACATGC
