# Supplementary material for: Diverse and tissue-enriched small RNAs in the plant pathogenic fungus, Magnaporthe oryzae
Source: BMC Genomics. 2011 Jun 2;12:288. doi: 10.1186/1471-2164-12-288 (PMC3132168; doi:10.1186/1471-2164-12-288)
Supplement: Additional file 5 — Distribution of mycelia small RNAs mapped to tRNAs. [file 1471-2164-12-288-S5.DOCX]

**Additional file 5** – Distribution of mycelia small RNAs mapped to tRNAs.

|  | Alignment^a^ | | |  | Read Count^b^ | | |  | Prorated^c^ | | |  | Features^d^ | | |
| --- | --- | --- | --- | --- | --- | --- | --- | --- | --- | --- | --- | --- | --- | --- | --- |
|  | Total | Sense | Antisense |  | Total | Sense | Antisense |  | Total | Sense | Antisense |  | Mapped | Total | Coverage |
| tRNA | 32008 | 26258 | 5752 |  | 5710 | 5623 | 89 |  | 4482 | 4447 | 35 |  | 356 | 361 | 99% |
| Ala | 1032 | 1032 | 1 |  | 379 | 379 | 1 |  | 310 | 310 | 0 |  | 15 | 15 | 100% |
| Arg | 352 | 352 | 1 |  | 224 | 224 | 1 |  | 209 | 209 | 0 |  | 15 | 16 | 94% |
| Asn | 253 | 253 | 1 |  | 131 | 131 | 1 |  | 91 | 91 | 0 |  | 8 | 8 | 100% |
| Asp | 2420 | 2420 | 1 |  | 848 | 848 | 1 |  | 528 | 528 | 0 |  | 12 | 12 | 100% |
| Cys | 43 | 43 | 1 |  | 43 | 43 | 1 |  | 30 | 30 | 0 |  | 3 | 3 | 100% |
| Gln | 560 | 560 | 1 |  | 238 | 238 | 1 |  | 175 | 175 | 0 |  | 8 | 8 | 100% |
| Glu | 2959 | 2957 | 4 |  | 579 | 579 | 2 |  | 306 | 306 | 0 |  | 11 | 12 | 92% |
| Gly | 1171 | 1171 | 1 |  | 256 | 256 | 1 |  | 207 | 207 | 0 |  | 21 | 22 | 95% |
| His | 479 | 479 | 1 |  | 128 | 128 | 1 |  | 70 | 70 | 0 |  | 5 | 5 | 100% |
| Ile | 127 | 127 | 1 |  | 70 | 70 | 1 |  | 68 | 68 | 0 |  | 10 | 10 | 100% |
| Leu | 2433 | 2433 | 1 |  | 863 | 863 | 1 |  | 473 | 473 | 0 |  | 15 | 15 | 100% |
| Lys | 3236 | 3228 | 10 |  | 646 | 646 | 2 |  | 322 | 321 | 1 |  | 13 | 13 | 100% |
| Met | 242 | 242 | 1 |  | 87 | 87 | 1 |  | 71 | 71 | 0 |  | 8 | 8 | 100% |
| Phe | 276 | 276 | 1 |  | 88 | 88 | 1 |  | 47 | 47 | 0 |  | 8 | 8 | 100% |
| Pro | 706 | 706 | 1 |  | 207 | 207 | 1 |  | 167 | 167 | 0 |  | 9 | 9 | 100% |
| SeC | 28 | 28 | 1 |  | 14 | 14 | 1 |  | 13 | 13 | 0 |  | 2 | 2 | 100% |
| Ser | 806 | 806 | 1 |  | 376 | 376 | 1 |  | 290 | 290 | 0 |  | 13 | 13 | 100% |
| Thr | 1428 | 1428 | 1 |  | 588 | 588 | 1 |  | 236 | 236 | 0 |  | 10 | 10 | 100% |
| Trp | 56 | 56 | 1 |  | 14 | 14 | 1 |  | 12 | 12 | 0 |  | 4 | 4 | 100% |
| Tyr | 105 | 105 | 1 |  | 21 | 21 | 1 |  | 16 | 16 | 0 |  | 5 | 5 | 100% |
| Val | 1473 | 1473 | 1 |  | 759 | 759 | 1 |  | 273 | 273 | 0 |  | 11 | 11 | 100% |
| Pseudo | 15322 | 9618 | 5706 |  | 1034 | 948 | 88 |  | 568 | 534 | 34 |  | 149 | 151 | 99% |
| Undet | 89 | 53 | 38 |  | 89 | 53 | 38 |  | 2 | 1 | 0 |  | 1 | 1 | 100% |

^a^ Alignment refers to the summation of small RNA alignments to any genomic feature.

^b^ Read Count represents the summation of distinct reads mapping to a given feature. Noteworthy the values for each genome feature are generally less than the sum of its sub-features due to the small RNAs mapping to multiple features (See “Material and Methods” for more details).

^c^ Prorated apportions the weight of any small RNA between alignments and features.

^d^ Features represent the proportion of genomic features mapped by small RNAs where mapped indicates the number of members for each genomic feature mapped by small RNAs among the total possible.
