# Supplementary material for: Diverse and tissue-enriched small RNAs in the plant pathogenic fungus, Magnaporthe oryzae
Source: BMC Genomics. 2011 Jun 2;12:288. doi: 10.1186/1471-2164-12-288 (PMC3132168; doi:10.1186/1471-2164-12-288)
Supplement: Additional file 6 — Distribution of appressoria small RNAs mapped to tRNAs. [file 1471-2164-12-288-S6.DOCX]

**Additional file 6** – Distribution of appressoria small RNAs mapped to tRNAs.

|  | Alignment^a^ | | |  | Read Count^b^ | | |  | Prorated^c^ | | |  | Features^d^ | | |
| --- | --- | --- | --- | --- | --- | --- | --- | --- | --- | --- | --- | --- | --- | --- | --- |
|  | Total | Sense | Antisense |  | Total | Sense | Antisense |  | Total | Sense | Antisense |  | Mapped | Total | Coverage |
| tRNA | 8749 | 8476 | 275 |  | 1989 | 1988 | 3 |  | 1636 | 1635 | 2 |  | 330 | 361 | 91% |
| Ala | 1996 | 1996 | 1 |  | 341 | 341 | 1 |  | 313 | 313 | 0 |  | 13 | 15 | 87% |
| Arg | 165 | 165 | 1 |  | 103 | 103 | 1 |  | 94 | 94 | 0 |  | 12 | 16 | 75% |
| Asn | 107 | 107 | 1 |  | 48 | 48 | 1 |  | 37 | 37 | 0 |  | 8 | 8 | 100% |
| Asp | 1589 | 1589 | 1 |  | 394 | 394 | 1 |  | 278 | 278 | 0 |  | 12 | 12 | 100% |
| Cys | 10 | 10 | 1 |  | 10 | 10 | 1 |  | 10 | 10 | 0 |  | 1 | 3 | 33% |
| Gln | 366 | 366 | 1 |  | 143 | 143 | 1 |  | 125 | 125 | 0 |  | 8 | 8 | 100% |
| Glu | 871 | 871 | 1 |  | 200 | 200 | 1 |  | 108 | 108 | 0 |  | 11 | 12 | 92% |
| Gly | 1068 | 1068 | 1 |  | 114 | 114 | 1 |  | 94 | 94 | 0 |  | 20 | 22 | 91% |
| His | 165 | 165 | 1 |  | 42 | 42 | 1 |  | 23 | 23 | 0 |  | 5 | 5 | 100% |
| Ile | 39 | 39 | 1 |  | 15 | 15 | 1 |  | 15 | 15 | 0 |  | 4 | 10 | 40% |
| Leu | 419 | 419 | 1 |  | 268 | 268 | 1 |  | 71 | 71 | 0 |  | 15 | 15 | 100% |
| Lys | 1044 | 1044 | 1 |  | 227 | 227 | 1 |  | 128 | 128 | 0 |  | 13 | 13 | 100% |
| Met | 110 | 110 | 1 |  | 58 | 58 | 1 |  | 40 | 40 | 0 |  | 7 | 8 | 88% |
| Phe | 9 | 9 | 1 |  | 3 | 3 | 1 |  | 1 | 1 | 0 |  | 8 | 8 | 100% |
| Pro | 94 | 94 | 1 |  | 39 | 39 | 1 |  | 33 | 33 | 0 |  | 9 | 9 | 100% |
| SeC | 0 | 0 | 0 |  | 0 | 0 | 0 |  | 0 | 0 | 0 |  | 0 | 2 | 0% |
| Ser | 214 | 214 | 1 |  | 85 | 85 | 1 |  | 69 | 69 | 0 |  | 11 | 13 | 85% |
| Thr | 753 | 753 | 1 |  | 215 | 215 | 1 |  | 136 | 136 | 0 |  | 10 | 10 | 100% |
| Trp | 20 | 20 | 1 |  | 5 | 5 | 1 |  | 4 | 4 | 0 |  | 4 | 4 | 100% |
| Tyr | 50 | 50 | 1 |  | 10 | 10 | 1 |  | 7 | 7 | 0 |  | 5 | 5 | 100% |
| Val | 77 | 77 | 1 |  | 46 | 46 | 1 |  | 45 | 45 | 0 |  | 10 | 11 | 91% |
| Pseudo | 287 | 16 | 273 |  | 12 | 11 | 3 |  | 7 | 5 | 2 |  | 143 | 151 | 95% |
| Undet | 2 | 1 | 2 |  | 2 | 1 | 2 |  | 0 | 0 | 0 |  | 1 | 1 | 100% |

^a^ Alignment refers to the summation of small RNA alignments to any genomic feature.

^b^ Read Count represents the summation of distinct reads mapping to a given feature. Noteworthy the values for each genome feature are generally less than the sum of its sub-features due to the small RNAs mapping to multiple features (See “Material and Methods” for more details).

^c^ Prorated apportions the weight of any small RNA between alignments and features.

^d^ Features represent the proportion of genomic features mapped by small RNAs where mapped indicates the number of members for each genomic feature mapped by small RNAs among the total possible.
