# Supplementary material for: Diverse and tissue-enriched small RNAs in the plant pathogenic fungus, Magnaporthe oryzae
Source: BMC Genomics. 2011 Jun 2;12:288. doi: 10.1186/1471-2164-12-288 (PMC3132168; doi:10.1186/1471-2164-12-288)
Supplement: Additional file 8 — End labelled oligonucleotides used for Northern blot. [file 1471-2164-12-288-S8.DOCX]

**Additional file 8** – End labeled oligonucleotides used for Northern blot.

| Primer ID | Sequence | Length (bp) |
| --- | --- | --- |
| 5’END tRNA^Thr^ (MGG_20128) | TACGAGTGATGCGCTCTACCACTGAGCTATACGGGC | 36 |
| 3’END tRNA^Thr^ (MGG_20128) | TGCCCGCACCCAGGATCGAACTAAGGACCTCATCAT | 36 |
| 5’END tRNA^Gly^ (MGG_20157) | GGCAACGATGGATTTTACCACTAAACCACTGATGC | 35 |
| 3’END tRNA^Gly^ (MGG_20157) | TGCATCAGCCGTGAATCGAACACGGGGCCCATCGA | 35 |
