# Supplementary material for: Diverse and tissue-enriched small RNAs in the plant pathogenic fungus, Magnaporthe oryzae
Source: BMC Genomics. 2011 Jun 2;12:288. doi: 10.1186/1471-2164-12-288 (PMC3132168; doi:10.1186/1471-2164-12-288)
Supplement: Additional file 10 — Correlation analysis of M. oryzae gene expression and number of mapped small RNAs. (A) Mycelia: gene expression (Y axis, NCBI GEO Accession GSE2716) in fungal tissue grown for 48 h (minimum medium) compared to the prorated values of small RNAs (X axis) isolated from fungus grown for 48 h in YeS medium. (B) Appressoria: gene expression data (12 h, GSE1945) compared to prorated values of small RNAs (16 h) from spores induced to form appressoria on a hydrophobic surface. [file 1471-2164-12-288-S10.PPTX]

## Slide 1
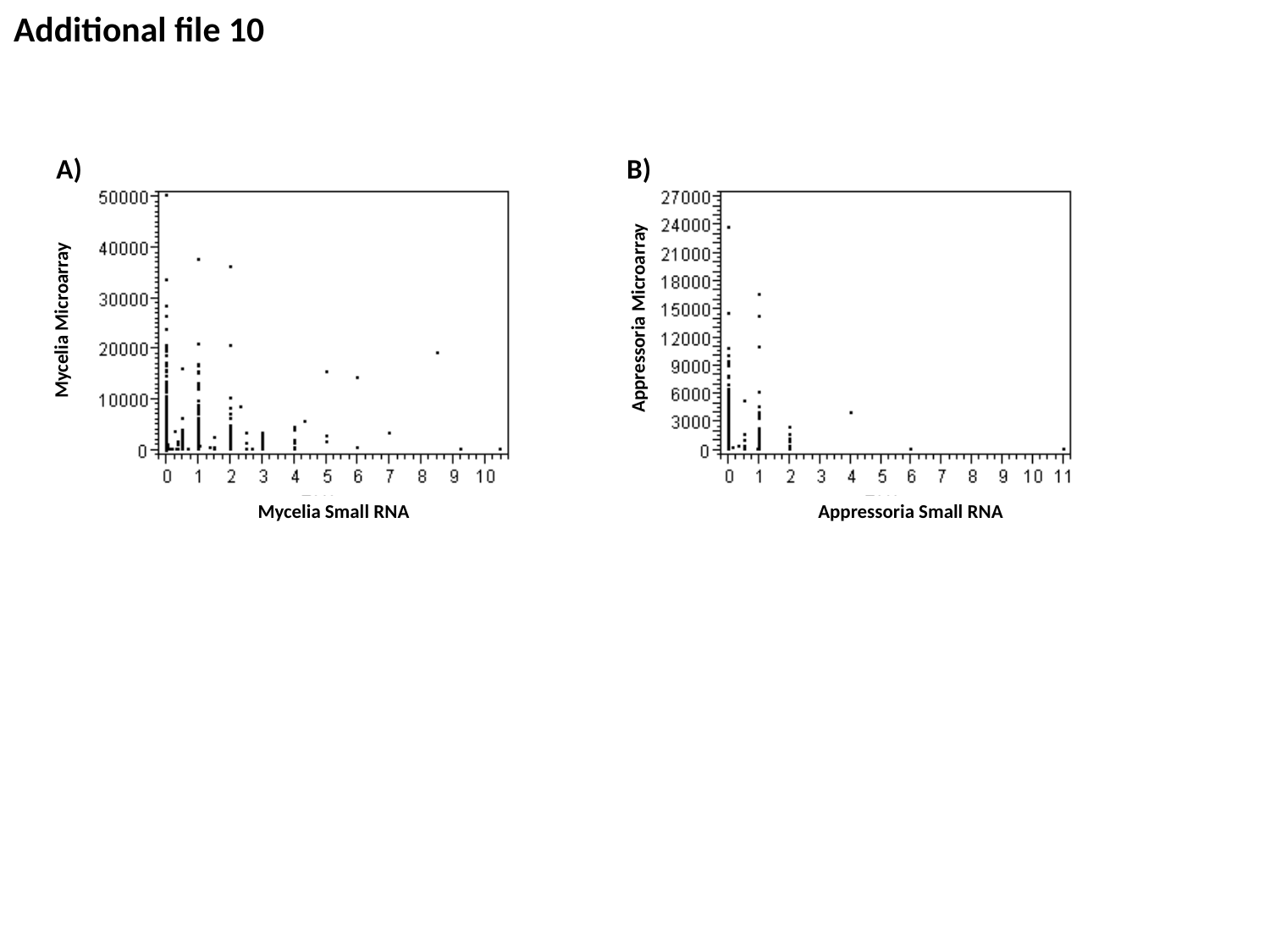

Additional file 10
A)
B)
Mycelia Microarray
Appressoria Microarray
Mycelia Small RNA
Appressoria Small RNA
