# Supplementary material for: Diverse and tissue-enriched small RNAs in the plant pathogenic fungus, Magnaporthe oryzae
Source: BMC Genomics. 2011 Jun 2;12:288. doi: 10.1186/1471-2164-12-288 (PMC3132168; doi:10.1186/1471-2164-12-288)
Supplement: Additional file 11 — Linkers and primers used for small RNA library preparation. [file 1471-2164-12-288-S11.DOCX]

**Additional file 11** – Linkers and primers used for small RNA library preparation.

| Oligos ID | Sequence |
| --- | --- |
| 3’ RNA/DNA adapter | /5Phos/rArGrGrCrC*rArCrArUrAGGCCGTCTTCAGCCGC*/3InvdT/ |
| 3’ adapter (RT) primer | GCGGCTGAAGACGGCCTATGT |
| 5’ RNA/DNA adapter (mycelia) | *rArGrCrArUrCrGrArGrUrCrGrGrCrCrUrUrGrUrUrGr*GrCrCrUrArCrUrGrG |
| 5’ adapter primer (mycelia) | AGCATCGAGTCGGCCTTGTTG |
| 5’ RNA/DNA adapter (appressoria) | *ATCGCCTCAGTCCGCATGCTAC*GGAGrGrArArUrUrC |
| 5’ adapter primer (appressoria) | ATCGCCTCAGTCCGCATGCTAC |

5Phos = phosphate group

idT = inverted deoxy thymidine modification

red letters = ribonucleotide sequence

black letters = deoxynucleotide sequence

italic letters = primers target
